# Unmasking Latent Inhibitory Connections in Human Cortex to Reveal Dormant Cortical Memories

**DOI:** 10.1016/j.neuron.2016.02.031

**Published:** 2016-04-06

**Authors:** H.C. Barron, T.P. Vogels, U.E. Emir, T.R. Makin, J. O’Shea, S. Clare, S. Jbabdi, R.J. Dolan, T.E.J. Behrens

**Affiliations:** 1 The Wellcome Trust Centre for Neuroimaging, Institute of Neurology, University College London, London WC1N 3BG, UK; 2 The Oxford Centre for Functional Magnetic Resonance Imaging of the Brain, University of Oxford, Oxford OX3 9DU, UK; 3 Centre for Neural Circuits and Behaviour, University of Oxford, Oxford OX1 3SR, UK

## Abstract

Balance of cortical excitation and inhibition (EI) is thought to be disrupted in several neuropsychiatric conditions, yet it is not clear how it is maintained in the healthy human brain. When EI balance is disturbed during learning and memory in animal models, it can be restabilized via formation of inhibitory replicas of newly formed excitatory connections. Here we assess evidence for such selective inhibitory rebalancing in humans. Using fMRI repetition suppression we measure newly formed cortical associations in the human brain. We show that expression of these associations reduces over time despite persistence in behavior, consistent with inhibitory rebalancing. To test this, we modulated excitation/inhibition balance with transcranial direct current stimulation (tDCS). Using ultra-high-field (7T) MRI and spectroscopy, we show that reducing GABA allows cortical associations to be re-expressed. This suggests that in humans associative memories are stored in balanced excitatory-inhibitory ensembles that lie dormant unless latent inhibitory connections are unmasked.

**Video Abstract:**

## Highlights

• Associative memories can be measured in human cortex following learning
• Over time the expression of these memories becomes silenced
• By reducing cortical GABA with brain stimulation these memories are re-expressed
• Cortical memories appear to be stored in balanced excitatory-inhibitory ensembles

## Teaser

Barron et al. show that otherwise silent cortical memories are unmasked in the human brain when the concentration of cortical GABA is reduced using brain stimulation. This suggests that memories are stored in cortex in balanced excitatory and inhibitory ensembles.

## Introduction

Local circuit level descriptions hold substantial promise for providing deep insights into neural function in health and disease. In contrast to the precise descriptions with which such mechanisms can be understood in animal experimentation, their effect on human cognition and psychiatric disorders can currently only be speculated about (Yizhar et al., 2011). This forces the assumption that neural mechanisms employed during simple tasks in animal models are directly parallel to those that support higher cognitive tasks of relevance to human life. It therefore remains a major challenge for contemporary neuroscience to develop noninvasive techniques that allow for investigation of neural circuit activity in humans. Here we designed an experiment for which we had strong predictions about the neural circuit level mechanism from data previously observed in animal models. We then asked whether we could use these circuit mechanisms to predict the precise macroscopic signals measured from the human brain.

The particular neural circuit mechanism observed in animal models and of particular interest for both cognitive function and dysfunction concerned the maintenance of detailed cortical balance. Synaptic input received by cortical neurons is balanced such that excitatory and inhibitory (EI) currents are precisely matched and stable firing preserved (Wehr and Zador, 2003, Okun and Lampl, 2008, Haider et al., 2006, Froemke et al., 2007, Xue et al., 2014, Shu et al., 2003). Both experimental and theoretical work suggests that this EI balance is critical for cortical processing, ensuring appropriate feature selectivity, gain control, temporal precision, and noise reduction of neuronal signaling (Wehr and Zador, 2003, Haider and McCormick, 2009, Isaacson and Scanziani, 2011). Failure to maintain cortical EI balance, via increased activity in excitatory neurons or reduction in inhibitory neurons, is hypothesized to give rise to the social and cognitive deficits observed in autism and schizophrenia (Lewis et al., 2005, Rubenstein and Merzenich, 2003, Yizhar et al., 2011).

Despite its importance, EI balance is disrupted during new learning, a process in which information is stored by modification of excitatory synaptic strengths (Hebb, 1949, Nabavi et al., 2014, Song and Abbott, 2001, Song et al., 2000). Experimental work in rodents and theoretical models now suggest that plasticity at inhibitory synapses may play an important role in restoring EI balance by allowing for inhibitory connections to precisely mirror their excitatory counterparts (D’amour and Froemke, 2015, Froemke et al., 2007, Vogels et al., 2011, Xue et al., 2014). Although detailed synaptic processes cannot be directly accessed in humans, here we sought to use these experimental and theoretical observations to predict the consequences of cortical rebalancing in the human cortex. We reasoned it should be possible to observe the macroscopic consequences of these microcircuit processes by combining approaches that index the similarity between subvoxel neuronal activity patterns using fMRI with techniques that manipulate and measure local cortical gamma-aminobutyric acid (GABA) concentration.

We hypothesized that when stimuli are paired together, their neuronal activity patterns should exhibit representational overlap at the subvoxel level, a consequence of the increase in strength of mediating excitatory connections. Furthermore, it should only be possible to observe this representational overlap during periods of EI imbalance, when excitatory connections that link the different stimulus representations dominate. Such EI imbalance has been reported immediately after learning, prior to inhibitory rebalancing (Froemke et al., 2007). We also reasoned that if cortical associative memories are maintained but rebalanced via inhibitory plasticity, it should be possible to induce a second period of EI imbalance to re-expose cortical memories. In line with previous investigations in both rodent motor cortex and songbird premotor cortex (Jacobs and Donoghue, 1991, Vallentin et al., 2016), we predicted that this second period of EI imbalance could be induced by downregulating the concentration of cortical GABA. This should lead to an increase in the representational overlap that underlies associative memories, in proportion to the induced change in GABA. Therefore, if associative memories are stored in balanced excitatory-inhibitory ensembles in the human cortex, cortical memories should lie dormant unless latent inhibitory connections are unmasked.

To test this prediction in the human brain, we first developed an index for the representational overlap between different subvoxel neural representations using fMRI repetition suppression. Using this index to provide a macroscopic signature of associative memories, we assessed representational overlap between paired stimuli immediately after learning. To assess the consequences of cortical rebalancing we then used fMRI repetition suppression to track changes in representational overlap over time, before combining this approach with anodal transcranial direct current stimulation (tDCS), a technique known to bring about a local reduction in cortical GABA (Kim et al., 2014, Stagg et al., 2009, Stagg et al., 2011). Using MR spectroscopy, we measured the accompanying change in GABA concentration in the region of cortex to which tDCS was applied.

We show that associated stimuli exhibit fMRI repetition suppression in cortex immediately after learning. The magnitude of this cross-stimulus suppression correlates with memory performance measured behaviorally, suggesting that it reflects expression of cortical memory. This cortical memory expression reduces over time and is absent the following day. Cortical memory can however be re-exposed by reduction in local GABA concentrations, induced using tDCS. The extent to which the memory is re-expressed occurs in proportion to the induced GABA reduction. By embedding memories in a spiking network model of memory formation (Vogels and Abbott, 2009, Vogels et al., 2011) and replicating each experimental step in silico, we show that these data are consistent with the balancing of memories via inhibitory synaptic plasticity in cortex.

## Results

### Measuring Associative Memories using fMRI Adaptation

**Figure 1:**
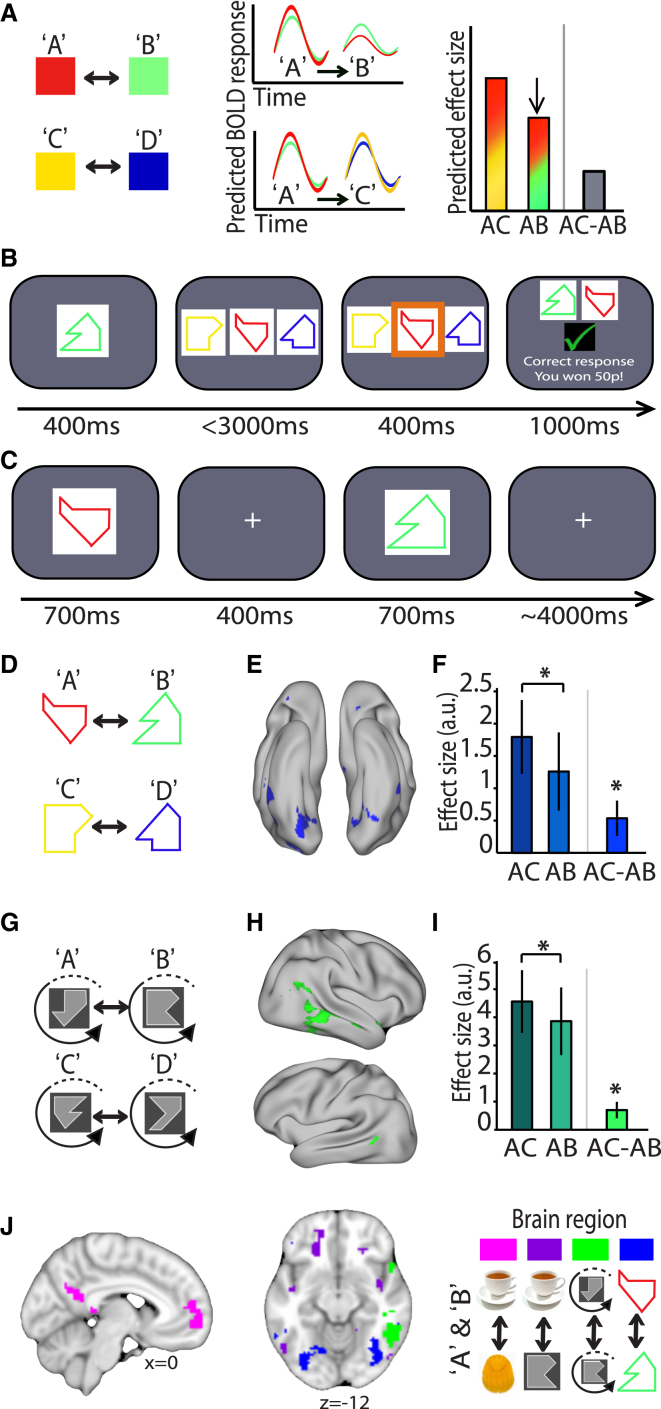
Indexing Cortical Associations in the Human Brain using Cross-stimulus Adaptation Immediately after Learning (A) Left: stimuli are associatively paired: A-B and C-D. Middle and right: due to repetition suppression, the predicted BOLD response to activation of associated but different stimuli, A followed by B, was reduced relative to consecutive unrelated stimuli, A followed by C. (B) Before entering the scanner, participants learned to associate pairs of stimuli using a three-alternative forced-choice task. On each trial, in response to a test shape, the participant had to select the associated stimulus from the full set. (C) During scanning, two stimuli were presented in short succession on each trial. (D) Using the task shown in (B), one set of participants learned to pair colored shapes (experiment 1), A with B and C with D. (E) Using the stimuli shown in (D), the BOLD response to consecutive presentation of two unrelated stimuli (AC, A followed by C) was contrasted against the BOLD response to consecutive presentation of two associated stimuli (AB, A followed by B): “unrelated” minus “associated,” and the contrast thresholded at p < 0.05 uncorrected for display purposes. (F) Parameter estimates (mean ± SEM) were extracted from an orthogonal ROI (see Table S1) in occipital and temporal cortices, for trials where stimuli were associated (AB, A followed by B) and trials where stimuli were unrelated (AC, A followed by C). The difference in parameter estimates for these two trial types (AC-AB, shown on the right) gave a significant cross-stimulus adaptation effect within this ROI (p = 0.043). (G) A second set of participants learned to associate rotationally invariant gray shapes (experiment 2), pairing A with B and C with D. (H) Using the stimuli shown in (G), the BOLD response to consecutive presentation of two unrelated stimuli (AC, A followed by C) was contrasted against the BOLD response to consecutive presentation of two associated stimuli (AB, A followed by B): “unrelated” minus “associated,” and the contrast thresholded at p < 0.05 uncorrected for display purposes. (I) Parameter estimates (mean ± SEM) were extracted from an orthogonal ROI (see Table S1) in right temporal cortex, for trials where stimuli were associated (AB, A followed by B) and trials where stimuli were unrelated (AC, A followed by C). The difference in parameter estimates for these two trial types (AC-AB, shown on the right) gave a significant cross-stimulus adaptation effect within this ROI (p = 0.024). (J) Cross-stimulus adaptation can be observed across cortex, in the anatomical regions that encode features specific to the associated stimuli. Blue region: colored shape associations as shown in (E). Green region: rotationally invariant stimulus associations as shown in (H).Purple region: stimuli associated with food reward (p = 0.032 within ROI). Pink region: associated imaginary food reward (p = 0.014 within ROI, see also Figure 4C of Barron et al., 2013).

To measure associative memories in the human cortex we needed to index neural representations that support the associated stimuli. With fMRI it is possible to use techniques that provide a measure of subvoxel neural representations. Here we used fMRI adaptation, a technique that relies on the fact that neurons show a relative suppression in their activity in response to repetition of a stimulus to which they are sensitive (Miller et al., 1991, Sawamura et al., 2006). While typically used to access the information content of a cell assembly via repetition of a single stimulus or stimulus feature (Grill-Spector et al., 2006, Krekelberg et al., 2006, Malach, 2012), more recently fMRI adaptation has been used to successfully index the representational similarity of two cell assemblies that each represent *different* stimuli (Barron et al., 2013). We hypothesized that we could use fMRI adaptation here to measure representational similarity of associated stimuli by contrasting the BOLD response to consecutive presentation of two associated stimuli against consecutive presentation of two unrelated stimuli (Figure 1A; Experimental Procedures).

We designed a series of pilot experiments to test this prediction and sought to identify a pair of stimuli which, when associated, gave adaptation in a brain region that could be later manipulated by extracranial stimulation inside the MRI scanner. We reasoned that cross-stimulus adaptation should be detectable in a cortical region predicted by the stimulus feature relevant for the association. For example, in recent data cross-stimulus adaptation between two associated imagined food reward was identified in the putative imagination network (Barron et al., 2013, Schacter et al., 2012). In three different low-N pilot experiments, participants learned to associate pairs of abstract visual stimuli using a behavioral training task (A was paired with B, and C with D) (Figure 1B). Stimuli were paired according to three different properties, each designed to engage a different cortical region (see Experimental Procedures). Immediately after learning, cross-stimulus fMRI adaptation between associated stimuli was assessed while participants performed an incidental “oddball” detection task, a task used to ensure that participants maintained attention to stimuli without being aware of adaptation measurements (Figure 1C; Experimental Procedures). Notably, we controlled for potential confounds introduced by expectation suppression (Summerfield et al., 2008) by ensuring that each pair of stimuli was presented equally often in a fully randomized order. To control for attentional effects, the BOLD response to consecutive presentation of two associated stimuli was then contrasted against consecutive presentation of two unrelated stimuli. To protect against concerns of multiple comparisons, we assessed cross-stimulus adaptation for each association by an independent regions of interest (ROI) analysis (Poldrack, 2007) (see Table S1 and Supplemental Experimental Procedures available online).

When the defining features for the association were shape and color (Figure 1D), significant adaptation between paired stimuli was observed in regions of occipital and temporal cortex (Figures 1E and 1F, t_8_ = 1.96, p = 0.043; cf. Table S1 for ROI specification), consistent with visual areas supporting the relevant features of this simple stimulus association. When participants associated abstract shapes in a rotationally invariant manner (Figure 1G), fMRI adaptation was observed within an anterior region of lateral occipital cortex (LOC), previously shown to represent rotational invariant features (Kourtzi et al., 2003) (Figures 1H and 1I, t_7_ = 2.41, p = 0.024; cf. Table S1 for ROI specification).When participants associated the same gray abstract shapes with an expected food reward, stimulus-reward pairs gave adaptation in lateral orbitofrontal cortex, a region known to respond to stimuli that predict specific reward (Klein-Flügge et al., 2013, Rudebeck and Murray, 2011) (Figure 1J; see Table S1 for ROI specification). Although the result from each of these pilot studies should not be considered in isolation due to the low number of subjects, the adaptation effect was reproducible across all four different studies (Table S1). Critically, these pilot studies provided a set of stimuli that could be used in combination with tDCS in a larger formal test below. In agreement with recent findings (Barron et al., 2013), these new results suggest that cross-stimulus adaptation can provide a measure of the representational similarity of paired stimuli, within the cortical region supporting features of the learned association.

### Cortical Associative Memories Are Silenced with Time

**Figure 2:**
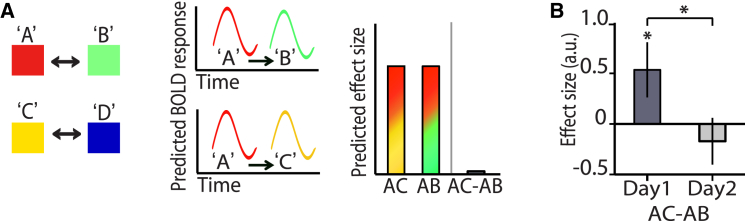
Cortical Associative Memories Are Silenced with Time (A) Left: example stimuli that were associatively paired: A-B and C-D. Middle and right: after inhibitory rebalancing had occurred, cross-stimulus adaptation between associated stimuli, A followed by B, was no longer predicted in the BOLD response as new inhibitory connections quench excitatory coactivation. Therefore activation of associated but different stimuli, A followed by B, was expected to be equivalent to activation of consecutive unrelated stimuli, A followed by C. (B) One set of participants (experiment 1) were scanned on a second occasion 24 hr after the initial scan and a significant reduction in cross-stimulus adaptation (measured with “associated” minus “not”) was observed across days (p = 0.045) (shown: mean ± SEM for each day).

Cross-stimulus adaptation therefore provides an index for cortical associative memory formation, and by implication, reflects the macroscopic consequences of modifications in excitatory interconnections. Having established this index, we went on to ask whether cross-stimulus adaptation could track subsequent predicted modifications in excitatory and inhibitory interconnections. Following the formation of new associative memories in anaesthetised rodents, cortical networks are rebalanced via inhibitory plasticity, strengthening inhibitory connections that lie between associated cell assemblies to quench excess excitatory activity (D’amour and Froemke, 2015, Froemke et al., 2007). These inhibitory rebalancing mechanism appear to have a time course of hours (Froemke et al., 2007).We therefore predicted that the consequence of inhibitory rebalancing upon cortical associations indexed here should be reflected in a reduction in representational similarity between associated cell assemblies, corresponding to a reduction in cross-stimulus adaptation (Figure 2A).

To test this prediction we performed a further pilot experiment. We re-scanned participants from one pilot experiment (colored shapes) on a second occasion, 24 hr after the initial session. A significant decrease in the magnitude of fMRI adaptation between associated stimuli was observed across days (Figure 2B, t_8_ = 2.37, p = 0.045; see also Figure S2A). This result is consistent with the idea that newly formed excitatory connections are subsequently balanced by proportional inhibitory connections that effectively mask access to the associative overlap of underlying cell assemblies. However, the same negative result would be predicted if the newly formed excitatory connections were subsequently depressed and the association forgotten. To disambiguate facilitation at inhibitory connections and depression at excitatory connections we adopted a more sophisticated approach.

### Predicted Consequences of Modulating GABA

If newly formed excitatory connections are subsequently balanced by proportional inhibitory connections, it should be possible to effectively re-expose these dormant associations by reducing cortical inhibition. Indeed, pre-existing lateral excitatory connections have previously been unmasked between motoric representations in neighboring M1 areas via pharmacological manipulation of GABA (Jacobs and Donoghue, 1991).

Applying this logic to the human brain we used a technique known to bring about a local reduction in cortical GABA, namely anodal tDCS. During and following cerebral direct current stimulation cortical excitability is enhanced as measured by local neuronal firing rates (Bindman et al., 1962) or remote motor evoked potentials (Nitsche et al., 2005). This enhancement is sustained after stimulation for minutes to hours (Bindman et al., 1962) via a protein synthesis dependent process (Nitsche and Paulus, 2000), contributing to its application to learning (Jacobson et al., 2012) and recovery from stroke (Hummel and Cohen, 2006). Evidence from direct spectroscopic measurements in vivo (Kim et al., 2014, Stagg et al., 2009, Stagg et al., 2011) and related electrical stimulation studies in vitro (Stelzer et al., 1987) suggest that this increase in excitability is caused by a reduction in available GABA concentrations (Stagg and Nitsche, 2011).

**Figure 3:**
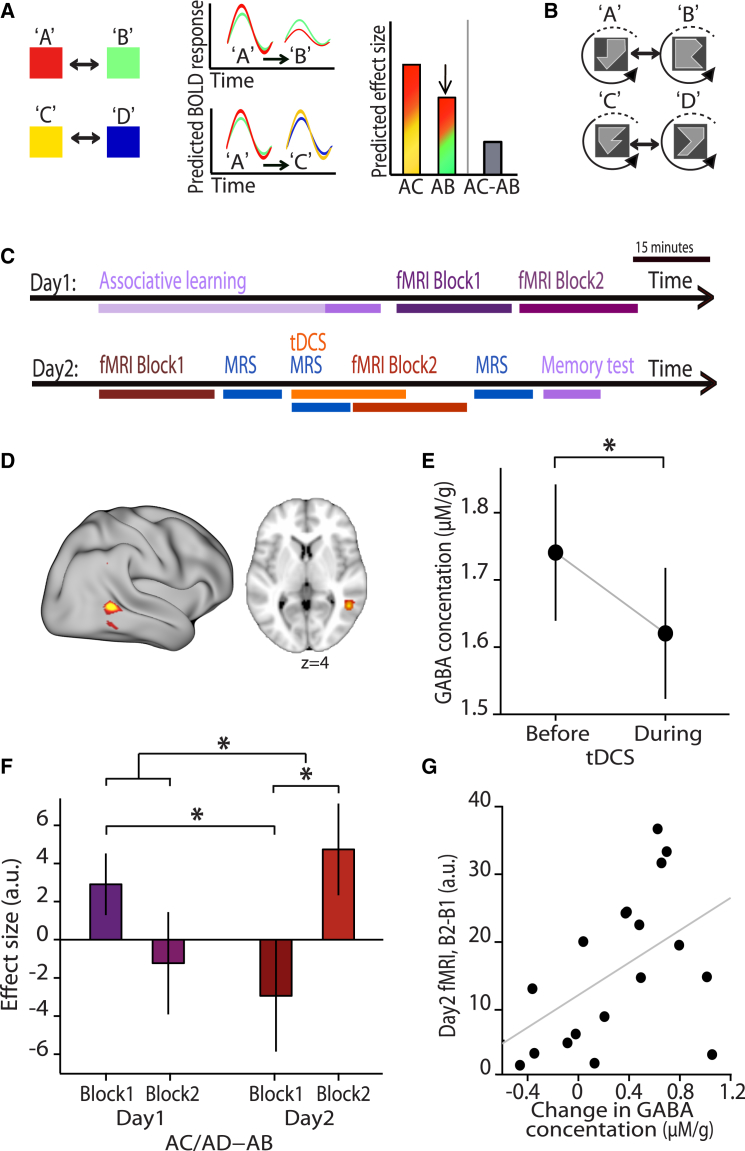
The Latent Cortical Associations Are Uncovered in the Human Brain via Local Modulation of GABA (A) Following downregulation of cortical GABA, cross-stimulus adaptation between associated stimuli, A followed by B, was once again predicted in the BOLD response relative to the control condition A followed by C. (B) Rotationally invariant shapes were used as the stimuli for the associative learning task (as in Figure 1G). (C) The protocol used to test for evidence of inhibitory rebalancing of cortical associations in the human brain. Participants completed the associative learning task shown in Figure 1B, before completing two fMRI task blocks. Returning 24 hr later, the fMRI task was repeated in conjunction with MRS and tDCS. The first fMRI task block was followed by a baseline MRS measurement. Twenty minutes of tDCS commenced, and a “during tDCS” MRS measurement simultaneously acquired. The second fMRI task block started half way through the tDCS session, followed by a final “post-task” MRS measurement. After exiting the scanner, participants were given a surprise memory test to check they still knew the paired associations. (D) The mean tDCS electrode location, with x-coordinate defined using the peak x-coordinates from Figure 1H. (E) By comparing MRS measurements acquired before and during tDCS (shown: mean ± SEM), a significant reduction in GABA concentration was observed (“baseline” stimulation minus “during” stimulation, p = 0.006). (F) B1 corresponds to block 1, and B2 to block 2. Parameter estimates were extracted to obtain a measure of cross-stimulus adaptation for each scanning block (mean ± SEM). As in Figure 1I, significant cross-stimulus adaptation was observed immediately after learning (Day1 B1, p = 0.044), and, as in Figure 2B, there was a significant reduction in cross-stimulus adaptation across days (Day1 B1 minus Day2 B1, p = 0.034). On day2, following tDCS, there was a significant increase in cross-stimulus adaptation (Day2 B2 minus Day2 B1, p = 0.006) and the interaction between this effect and day 1 was also significant (day ^∗^ block: [(Day2 B2 minus Day2 B1) minus (Day1 B2 minus Day1 B1)], p = 0.010). (G) The change in GABA concentration before versus during tDCS correlated with the change in cross-stimulus adaptation from Day2 B1 to Day2 B2 (with effects due to glutamate removed, r_17_ = 0.486, p = 0.041).

Here we applied anodal tDCS to a region of cortex where cross-stimulus adaptation was measured immediately after learning but had since reduced with time. This led to the following two predictions. First, a tDCS-induced reduction in cortical GABA should selectively *increase* fMRI adaptation between associated versus unrelated stimuli, owing to stronger excitatory connections mediating the associative cell-assemblies (Figure 3A). Second, this predicted re-emergence of associative memories should be proportional to the tDCS-induced reduction in GABA.

### Manipulating GABA to Re-expose Dormant Cortical Memories

To test these predictions we applied tDCS in conjunction with our fMRI adaptation paradigm. In parallel, we quantified the concentration of GABA using magnetic resonance spectroscopy (MRS), a technique used in vivo to measure the relative concentration of target metabolites in the brain. To achieve near simultaneity in fMRI adaptation measurements and MRS quantification of GABA concentration, we used 7T MRI with its accompanying benefits of higher signal-to-noise ratio (SNR) and chemical shift dispersion. From our three pilot experiments, the protocol with rotationally invariant shapes was the most appropriate, because it produced cross-stimulus adaptation in an accessible brain region for tDCS.

As in pilot experiments, participants first learnt to pair the rotationally invariant shapes (Figure 3B). We then measured cross-stimulus adaptation in two subsequent fMRI sessions (as in Figure 1C). When participants returned 24 hr later, we combined two additional fMRI sessions with the MRS and tDCS protocol (Figure 3C). The anodal tDCS electrode was placed over the occipital-temporal location previously shown to adapt to associated, rotationally invariant shapes (Figure 1H; mean anodal electrode location, Figure 3D; see also Figure S1). The cathode was placed over the contralateral supraorbital ridge. MRS measurements were taken from a 2 × 2 × 2 cm^3^ voxel, approximately centered underneath the anode (Figure S1C), and could be rapidly acquired before, during and after tDCS (for example spectra see Figures S1A and S1B; see Experimental Procedures for further details).

As predicted, we found a significant decrease in MRS-quantified GABA concentration during tDCS compared to baseline (“baseline” minus “during tDCS,” Figure 3E, t_17_ = 2.81, p = 0.006). This reduction was not sustained after the subsequent task (Figure 5A, t_17_ = 1.20, p = 0.123). The only other metabolite (n = 19) to show a change in concentration at the same significance level (p < 0.05) was glutamate, which had significantly increased in concentration (Figure 5B, t_17_ = 2.22, p = 0.020), but only at a later time point after the task.

We then asked whether the tDCS-induced reduction in GABA was accompanied by an increase in cross-stimulus adaptation, reflecting the increase in expression of cortical associations that would be predicted by unmasking previously inhibited cortical associations. The analysis was tightly constrained by our prior hypotheses and the experimental design: the increase in cross-stimulus adaptation was expected directly underneath the anodal tDCS electrode, at the mean cortical depth reported in our pilot data (Figure 1H). Parameter estimates for our regressors of interest were therefore extracted from the unbiased peak tDCS electrode location (peak of Figure 3D) at the predicted cortical depth. This precise prediction could only be made due to the pilot experiments, reported in detail above.

If cortical memories are expressed only during periods when cortical associations can be described as being free from inhibition or in EI imbalance, it should be possible to measure cross-stimulus adaptation during block 1 on the first day (before balancing) and block 2 on the second day (after unbalancing), but not during block 1 on the second day (after balancing). The critical test was therefore a two-way ANOVA (day ^∗^ block). Notably this ANOVA has in-built controls for block and day. This test revealed a significant interaction (Figure 3F, day ^∗^ block, F_1,64_ = 8.05, p = 0.010), suggesting that the expression of associative memories was restored during tDCS application. The directionality of this interaction was verified using post hoc t tests, which first showed a replication of our previous findings (Figures 1I and 2B), with significant cross-stimulus adaptation in the first fMRI session (Figure 3F, “Day1 B1,” t_20_ = 1.80, p = 0.044; see also Figures S2F and S3G). Furthermore, we again observed a significant decrease in cross-stimulus adaptation by the first session of Day 2 (Figure 3F, “Day1 B1” > “Day2 B1,” t_20_ = 1.93, p = 0.034; see also Figure S2F), but not the second session of Day1 (Figure 3F, “Day1 B1” > “Day1 B2,” t_20_ = 0.85, p = 0.797), suggesting that the cortex rebalanced after 24 hr. Critically, after application of tDCS, the cross-stimulus adaptation returned (Figure 3F, “Day2 B2” > “Day2 B1,” t_20_ = 3.08, p = 0.006; see also Figures S2E–S2G), confirming that adaptation was greater during periods of putative EI imbalance (Figure 3F, Interaction [“Day2 B2” > “Day2 B1”] – [“Day1 B2” > “Day1 B1”]; t_20_ = 2.84, p = 0.010; see also Figures S2D and S2F). These results demonstrate that dormant neuronal relationships can be revealed by local reduction of GABA, suggesting that expression of cortical associative memories is controlled by selective inhibitory connections.

### Re-exposure of Otherwise Dormant Memories Is Predicted by the Change in GABA

To further establish the relationship between the change in GABA concentration and re-expression of an associative memory, and to assess the specific contribution of GABA, we measured the correlation between the fMRI adaptation effect and the change in GABA concentration across the population. To maximize sensitivity across the group, parameter estimates for the adaptation effect were extracted from individual-specific regions, defined by the individuals’ peak interaction effect (see Supplemental Experimental Procedures). This allowed us to identify the strongest recovery in fMRI adaptation in each individual. The increase in cross-stimulus adaptation observed after tDCS on day 2 significantly correlated with the change in GABA observed during tDCS (Figure 3G, r_17_ = 0.486, p = 0.041, after accounting for changes in glutamate, see also Figures S3A–S3C). Importantly, there was no significant correlation between these adaptation effects and any of the other 18 metabolites measured with MRS, including glutamate (see Figures S3D–S3G). These results provide further independent statistical evidence that dormant memories can be re-expressed in cortex by local reductions in GABA, and demonstrate that the effect is specific to GABA among the 19 metabolites that we could measure with spectroscopy.

The variation in GABA concentration observed across participants is similar to previous studies that compared real versus sham tDCS (Stagg et al., 2009, Stagg et al., 2011). By virtue of the precise quantitative predictions made about the relationship between fMRI adaptation and GABA concentration, it was not necessary to include a separate sham condition here. The range of inter-individual variation provided a more stringent framework within which to test our hypotheses. In effect, fMRI adaptation measured from participants with a lower change in GABA parametrically controlled for that measured from participants with a higher change in GABA.

### Behavior Predicts Cross-stimulus Adaptation

**Figure 4:**
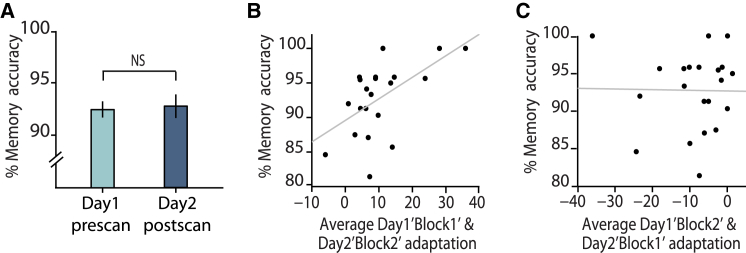
Memory Accuracy Predicts Cross-stimulus Adaptation (A) There was no significant difference between participants’ accuracy on the associative learning task performed on day1 and the surprise memory test performed after scanning on day2 (p = 0.821) (shown: mean ± SEM for each day). (B) During periods of EI imbalance (Day1-block1 and Day2-block2), the average cross-stimulus adaptation significantly correlated with memory performance on the surprise memory test (r_20_ = 0.57, p = 0.007). (C) During periods of EI balance (Day1-block2 and Day2-block1), the average cross-stimulus adaptation did not correlate with memory performance on the surprise memory test (r_20_ = 0.016, p = 0.946).

By unmasking previously silent cortical associations, our data suggest that although the expression of cortical associations reduces over time, learned associations may be stored as balanced ensembles of excitatory and inhibitory connections rather than subject to depression at excitatory synapses. This is further supported by analysis of participants’ behavior during a surprise memory test performed after the final scanning session. Memory accuracy did not differ from performance at the end of the pre-scan training on day 1 (accuracy on last block day1 versus accuracy on day 2 (dark mauve in Figure 3C) (Figure 4A, t_20_ = 0.94, p = 0.821). Remarkably, this measure of behavioral performance could be used to predict the neural index for the expression of cortical memories, measured using cross-stimulus adaptation. Memory accuracy on the surprise test correlated with the average cross-stimulus adaptation for task sessions during putative imbalance (day1-block1, day2-block2) (Figure 4B, r_20_ = 0.57, p = 0.007; see also Figures S3H and S3I), but not with the average cross-stimulus adaptation during putative periods of balance (day1-block2, day2-block1) (Figure 4C, r_20_ = 0.016, p = 0.946; see also Figures S3J and S3K). The correlation between memory accuracy and the day ^∗^ session interaction of cross-stimulus adaptation showed a similar trend (r_20_ = 0.41, p = 0.069). This result suggests that memory performance can be used to predict the magnitude of cortical cross-stimulus adaptation during periods of reduced cortical GABA.

### Cortical Excitability, and GABAergic and Glutamatergic Spectroscopy Measurements

**Figure 5:**
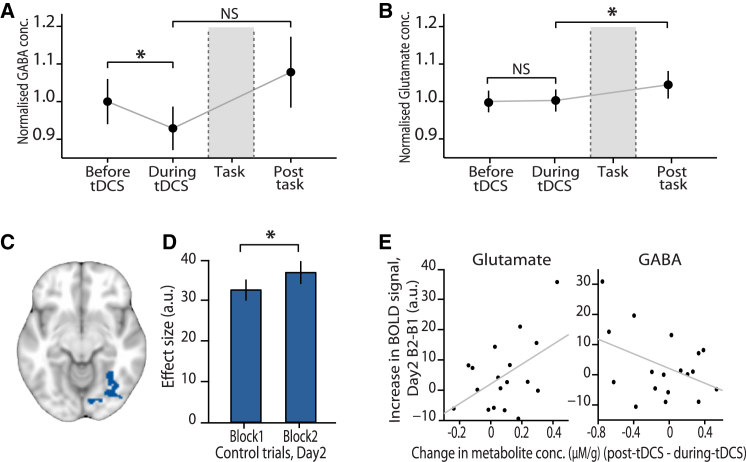
Cortical Excitability and Changes in GABA and Glutamate Concentration (A) The concentration of GABA for each MRS acquisition, averaged across the group (mean ± SEM). As shown in Figure 3E, a significant reduction in GABA concentration was observed when comparing MRS measurements acquired before and during tDCS (p = 0.006). There was no significant difference between these GABA concentration measurements and the GABA concentration measured after the fMRI task block (p = 0.114). (B) The concentration of glutamate for each MRS acquisition, averaged across the group (mean ± SEM). There was no significant difference between glutamate concentration measured before versus during tDCS (p = 0.872). However, there was a significant increase in glutamate after the final fMRI task block (p = 0.020). (C) The region of interest used to assess changes in raw BOLD following application of tDCS. To avoid confounding our analysis with adaptation effects this ROI was defined from the average BOLD response to pairs of unrelated stimuli across all task blocks (see Supplemental Experimental Procedures). (D) Parameter estimates (mean ± SEM), extracted from the ROI shown in (C), revealed a significant increase in the raw BOLD response to nonadapting stimuli following application of tDCS (block2 – block1: p = 0.043). (E) The increase in BOLD response, shown in (D), was predicted by the post-task increase in cortical excitability, measured using MRS (change in glutamate concentration contrasted with change in GABA concentration using multiple regression: p = 0.024). This result is illustrated here by the positive correlation between the change in BOLD and post-task change glutamate concentration (r_17_ = 0.488, p = 0.0398, with effects due to GABA removed) (left), and the negative trend between the change in BOLD and the post-task change in GABA concentration (r_17_ = −0.424, p = 0.080, with effects due to glutamate removed) (right).

It is notable that the tDCS-induced GABA change led to an increase in adaptation, and therefore *reduced* signal in trials with paired stimuli compared to controls. Net increases of cortical excitability might be expected to lead to a general *increase* in measured BOLD signal. To test this, we extracted the BOLD response for the control trials alone. Indeed, the response to control trials showed a small increase following tDCS (Figures 5C and 5D, Day2 block2 – block1: t_20_ = 1.81, p = 0.043; see Supplemental Experimental Procedures for ROI specification). While this general increase did not correlate with the GABA reduction observed during tDCS (r_17_ = −0.117, p = 0.643, after accounting for changes in glutamate), it was predicted by the change in spectroscopic measurements over the course of the task. Notably, the change in glutamate concentration over the final task (post-task – during-tDCS) positively predicted the change in BOLD response (Figure 5E, multiple regression, see Supplemental Experimental Procedures: t_17_ = 2.17, p = 0.022). The equivalent change in GABA concentration negatively predicted the change in BOLD response (Figure 5E, multiple regression, see Supplemental Experimental Procedures: t_17_ = 1.81, p = 0.044). These opposite effects of glutamate and GABA measurements lead to the estimated change in cortical excitability (glutamate contrasted with GABA) predicting the observed change in BOLD fMRI in the control trials (multiple regression, see Supplemental Experimental Procedures: t_17_ = 2.13, p = 0.024), lending further credence to the specificity of the spectroscopic measurements.

### Simulation using a Neural Network Model

**Figure 6:**
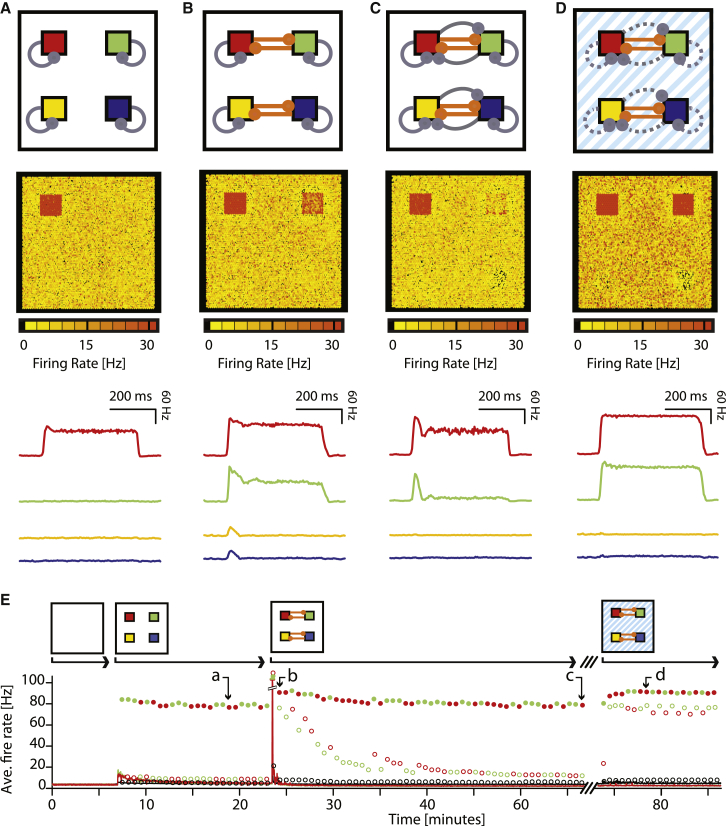
Neural Network Model Showing How Latent Cortical Associations Can Be Uncovered by Downregulating the Efficacy of Inhibitory Neurons (A–D) Four snapshots of recurrent network activity in response to stimulating one of four embedded cell assemblies. In the first row, each panel features a schematic of the parameter conditions of the network. The assemblies are pictured as colored squares. Excitatory and inhibitory connections are drawn in orange and gray, respectively. The second row shows the average firing rate over 1 s of every excitatory neuron in the network, assembled on a square grid. The third row visualizes the average firing rate of all excitatory neurons in each (red, green, yellow, or blue colored) assembly, averaged over 5 trials. (A) In the initial, balanced state, activation of the upper left (red) cell assembly leads to high firing rates in the activated neuron group, but not in other neurons (cf. Figure S4A). (B) After excitatory connections between associated cell-assemblies were selectively enhanced, the activation of the same assembly coactivates the associated green cell-assembly. (C) After disynaptic inhibition has been strengthened to balance the surplus excitation, the stimulation no longer resulted in coactivation of the associated green cell assembly. (D) Reducing the efficacy of all inhibitory synapses in the balanced network restored coactivation of the associated cell assembly (green) in response to driving the red cell assembly. (E) Complete simulation of all stages of the protocol (A) through (D) in 80 min and accordingly adjusted learning rate η. Solid lines show the average activity of the red and green cell assemblies over 2 s, and the activity of all background neurons is plotted in black. Circles show the average firing rate of red and green assembly neurons when they are stimulated (solid circles) or when the other assembly is stimulated (open circles), at 40 s intervals. Open black circles show the firing rates of un-stimulated background neurons during stimulations. The simulation begins with a naive network without assembly structure, firing at 5 Hz. After four cell assemblies are introduced (t = 7 min) the firing rate of assembly and background neurons increases, but inhibitory synaptic plasticity re-stabilizes network activity at 5 Hz. Red and the green cell assemblies can be individually activated, as shown in (A). When “associative” connections *between* the red and the green, and the blue and yellow (data not shown) cell assemblies are introduced (t = 23.5 min), high firing rates (maximum 136 Hz) of the unstimulated network are adjusted over the course of several minutes, but the associated cell assemblies coactivate in response to stimulation of either assembly, as shown in (B). Over time, inhibitory plasticity refines the disynaptic inhibitory inputs to each assembly so that coactivation between associated assemblies is reduced, as shown in (C). By reducing the efficacy of all inhibitory synapses, as thought to occur during tDCS (t = 74 min), the coactivation between associated cell assemblies is recovered, as shown in (D).

The *selective* re-expression of previously dormant cortical associations was observed by combining a nonspecific tDCS-induced reduction of GABA with representational fMRI. Individual cortical associations could therefore be released and measured despite the global reduction in GABA. These macroscopic observations are the logical consequence of rebalancing the cortical circuit, where balanced excitatory-inhibitory ensembles are maintained via inhibitory plasticity. To further illustrate how these observations can be considered the consequences of circuit level synaptic modifications, we refined a set of previously published neural network models (Vogels et al., 2013, Vogels and Abbott, 2009) to incorporate the experimental protocol presented above. In the network model, we included four cell assemblies to represent independent and nonoverlapping representations of the four stimuli (A:D), that were balanced by local inhibition (Vogels et al., 2011) (Figures 6A and S4A). Each cell assembly could be activated individually by selectively reducing the efficacy of the relevant local interneurons. To simulate the consequences of learning new associations, we selectively strengthened excitatory connections *between* pairs of cell assemblies (Nabavi et al., 2014) (see Supplemental Experimental Procedures). Immediate subsequent activation of one cell assembly (e.g., red) resulted in co-activation of its associated pair (e.g., green, Figures 6B and S4B). Over time, inhibitory plasticity balanced the surplus excitation in each assembly, restoring balance to the network (Figures 6C, 6E, and S4C). Despite strong excitatory connections between assemblies, coactivation was effectively silenced by the proportionally strengthened disynaptic inhibitory connections.

Our model thus qualitatively reproduced the key features of the experimental results: immediately after learning, paired cell assemblies within the network coactivated and therefore had overlapping representations (Figures 1, 3F, and 6B; see also Figure S5B); these paired representations were separated again when inhibitory rebalancing occurred (Figures 2, 3F, and 6C; see also Figure S5B). In line with previous work (Litwin-Kumar and Doiron, 2014, Zenke et al., 2015), such separation of stable memories could not be achieved if we instead used homeostatic scaling to stabilize network activity in the absence of inhibitory plasticity (Figure S5C).

Having thus embedded two hidden associative memories in the network, we then tested if these associations could be re-exposed via a network-wide manipulation of inhibition. We downregulated the efficacy of all inhibitory synapses by 15%, a percent reduction inspired by previous tDCS-induced changes in cortical GABA concentration (Kim et al., 2014, Stagg et al., 2009). Coactivation of the previously paired cell assemblies was recovered when either assembly was stimulated individually (Figures 6D and 6E; see also Figures S4D, S5, and S6), and similar results were observed when inhibition was reduced by approximately 8%, up to approximately 40% (Figure S6). Notably, despite the global nature of the manipulation, the resulting EI imbalance led to only moderate changes in the background activity but substantially amplified the effect of excitatory connections between associated cell assemblies. By contrast, when the network was stabilized with homeostatic scaling of the excitatory synapses, instead of inhibitory synaptic plasticity, it did not show these effects. Rather it produced network wide instabilities and assembly “latching,” i.e., uncontrollable serial activation of random assemblies (Figure S5C). These modeling results illustrate how a general reduction in network inhibition may be sufficient to selectively expose associations between otherwise balanced cell-assemblies, and thus qualitatively resemble the selective unmasking of otherwise dormant cortical memories observed in humans following application of tDCS (Figures 3F and 3G).

## Discussion

We have shown that otherwise dormant associative memories can be re-expressed in human cortex by reducing the concentration of cortical GABA using anodal tDCS. This was made possible by first establishing an index for associative memories in the human cortex using fMRI adaptation. Immediately after learning, adaptation between associated stimuli was observed in proportion to memory performance measured behaviorally. By tracking this index for associative memories across time, we show that adaptation between associated stimuli is significantly reduced after 24 hr, but can be recovered by reducing the concentration of cortical GABA using tDCS. These results suggest that associative memories lie dormant in human cortex but can be selectively expressed following changes in cortical excitability.

By combining multiple imaging techniques with brain stimulation, these data provide a macroscopic readout of cortical memory formation that reflects the consequence of underlying circuit level processes. Taking each finding in turn, it is possible to infer the nature of these underlying circuit level processes from related data in animal models. For example, the neural circuit mechanisms that accompany fMRI adaptation between recently associated stimuli may be inferred from the following two observations in animal models. First, associative learning is accompanied by modifications at excitatory synapses which increase co-activation between associated cell assemblies (Nabavi et al., 2014). Second, neuronal adaptation is observed in single-unit recording following consecutive presentation of different stimuli to which a neuron is sensitive (Sawamura et al., 2006). fMRI adaptation between recently associated stimuli may therefore be interpreted as an index for co-activation between associated cell-assemblies, the consequence of excitatory plasticity that occurs during learning.

Similarly, the observed reduction in adaptation across time, but subsequent recovery following application of tDCS may also be interpreted using neural circuit level processes measured in animal models. Of particular relevance is the observation that modifications at excitatory synapses are accompanied by complementary changes at inhibitory synapses in rodent auditory cortex, which rebalance cortex over a time course of hours (D’amour and Froemke, 2015, Froemke et al., 2007). Following memory formation, EI balance may therefore be restored by precisely complimenting excitatory connections with inhibitory replicas, or antimemories. This is thought to be important in providing stable storage for multiple individual memories since antimemories can prevent spontaneous memory activation, an effect known as latching in the modeling literature (Linkerhand and Gros, 2013, Abeles et al., 1995, Litwin-Kumar and Doiron, 2014, Zenke et al., 2015). Pharmacological manipulation of rodent motor cortex suggests that formation of antimemories may be a common feature of cortex more generally since relief of inhibition in this cortical region also reveals latent intracortical excitatory connections (Jacobs and Donoghue, 1991).

In light of these data, we infer that the observed reduction in fMRI adaptation after 24 hr reflects the consequence of modifications at inhibitory synapses which act to restore cortical EI balance following associative learning. Recovery of adaptation during tDCS-induced reduction in cortical GABA demonstrates that selective inhibitory connections are otherwise responsible for silencing adaptation between associated stimuli. Our data are therefore consistent with the suggestion that cortical associations are stored as balanced excitatory and inhibitory ensembles which remain silent unless EI balance is disrupted.

The formation of inhibitory replicas of memories, or antimemories, via inhibitory plasticity likely complements other homeostatic mechanisms such as synaptic scaling (Litwin-Kumar and Doiron, 2014, Turrigiano and Nelson, 2004, Turrigiano et al., 1998, Zenke et al., 2015) where, following Hebbian learning, cortical stability can be maintained via normalization of all excitatory synapses in the network (Turrigiano, 2008). In network modeling, homeostatic plasticity alone is not sufficient to explain the phenomenon of memory embedding or, more importantly, retrieval via GABA decrease (Zenke et al., 2015). Given these difficulties, it seems unlikely that synaptic scaling alone could account for the data. Furthermore it does not provide a simple explanation for the empirical observations. For example, a difference in cross-stimulus adaptation between associated and nonassociated cell assemblies is not maintained across time as would be predicted by synaptic scaling. By contrast, the explanation provided for the data by inhibitory plasticity can fully account for the empirical observations and provides a parsimonious description of the data.

Although we are unable to experimentally verify this interpretation of the data, we consider our approach nonetheless important. We have shown how a multimodal noninvasive approach can be used to obtain macroscopic measurements of human brain activity which reflect the consequence of neural circuit level processes. By considering microcircuit processes previously observed in animal and theoretical models, we used a highly constrained experimental design to generate precise predictions. From the data it was therefore possible to infer plausible neural circuit level processes that contribute to the observed macroscopic signal. This approach may provide a foundation for inferring subvoxel neural mechanisms that cannot be directly imaged in humans yet are likely to underlie neurological and pathological disease.

Indeed, failure to maintain balance in cortex has been hypothesized as a substrate for pathophysiological consequences observed in autism, epilepsy and schizophrenia (Lewis et al., 2005, Rubenstein and Merzenich, 2003, Yizhar et al., 2011). For example, elevating excitation in rodents introduces social deficits (Yizhar et al., 2011), while pharmacological suppression of inhibition rapidly leads to epileptic-like spread of synchronized excitation to distant cortical sites (Chagnac-Amitai and Connors, 1989). Furthermore, when the balance of excitation and inhibition is not properly maintained in a simulated neural network, the model exhibits effects that can be related to hallucinatory and delusional symptoms (Vogels and Abbott, 2007). Given the proposed contribution of EI imbalance to this range of psychiatric disorders, it is critical that we develop tools in humans that allow for the underlying neural mechanisms to be uncovered.

While we have focused this investigation on the formation of new associations in sensory regions of cortex, the question of how balanced associative information is recalled remains pertinent. Interactions between different brain regions and modalities of stored information may play a critical role. Here, to avoid confounding our measure of cross-stimulus adaptation, it was only possible to test memory behaviorally at the very end of the experiment, giving a measure for memory accuracy only when the memory had arguably been released following application of tDCS. It was therefore not possible to explore the nature of memory recall following rebalancing. Nevertheless, we hypothesize that recall may involve the release from balance of stored information. The advantage of maintaining inhibitory replicas of memories is then readily apparent: multiple memories can be stored stably, but each memory can be easily and selectively recalled through disinhibition. By altering the strength of inhibition, it may therefore be possible to gate excitability of particular cortical circuits. Indeed, recent optogenetic manipulation of rodent cortex and hippocampus suggests that the cortex provides a sufficient store for memories and hippocampus may serve as the cortical gate (Cowansage et al., 2014). Having demonstrated how circuit level activity may be indirectly indexed in the human brain, we here provide an example protocol from which to start investigating circuit level descriptions of memory recall and other cognitive functions, providing a potential means to reveal the neural computations that contribute to human cognition.

## Experimental Procedures

### Participants

Fifty-three healthy volunteers participated in the study (see Table S1 for summary; experiment 1, “colored shapes”: n = 9, mean age of 22.3, 5 females; experiment 2, “rotationally invariant shapes (3T)”: n = 9, mean age of 24.8, 7 females; experiment 3, “stimulus-reward”: n = 10, mean age of 21.3, 6 females; experiment 4, “rotationally invariant shapes (7T)”: n = 25, mean age of 22.7, 11 females). Experiments 1–3 were approved by the University College London ethics committee (reference number 3450/002), and experiment 4 was approved by the Oxford University ethics committee (reference number MSD-IDREC-C2-2013-20). All participants gave informed written consent.

In experiments 2 and 4, one participant was excluded due to sleepiness during the scanning session, verified respectively using an eye tracker and personal report. In experiment 4, an additional three participants moved more than 5 mm during the first scanning session and were excluded from data analyses involving fMRI measurements from this session.

### Behavioral Training

Four different stimuli were presented to the participant: A, B, C, and D, with a fully factorized randomization of stimulus allocation across participants. In experiment 1, stimuli were colored shapes (Figure 1D). In experiments 2 and 4, stimuli were rotationally invariant gray shapes (Figures 1G and 3B), which were observed in one of four possible rotations, with each rotation separated by 90°. In experiment 3, stimuli were gray shapes and food reward (Figure 1J). The rotationally invariant gray shapes used in experiments 2 and 4 included four different shapes each of which could be observed in one of four possible orientations.

Participants were trained to pair these stimuli (A with B, and C with D), using a three-alternative forced-choice task (Figure 1B). On each trial, one of the four stimuli was shown for 400 ms before all three remaining stimuli were presented in randomized positions across the screen. Participants were instructed to press the button associated with the correct stimulus’ position, as quickly and accurately as possible. Accurate and fast responses were rewarded with 50 pence, with the threshold for a fast response titrated to the participants mean reaction time. Ten percent of trials were randomly selected at the end of each task block and the participant received the sum total reward from these trials. Participants were required to continue with this stimulus-item learning task until their average reaction time per block approached 700 ms with 90% accuracy.

### fMRI Task, Data Acquisition

In all four experiments, fMRI measurements were acquired while participants viewed a series of visual stimuli, presented via a computer monitor projected onto a screen. The visual stimuli comprised the four stimuli used in the training task, A, B, C, and D, except in experiment 1, where stimulus D was replaced by a novel stimulus, E (see Table S1).

On each trial two stimuli were presented consecutively for 700 ms each, with an interstimulus interval of 400 ms (Figure 1C). The intertrial interval was selected from a truncated gamma distribution (experiments 1–3) or uniform distribution (experiment 4) with mean of 4 s. To control for potential confounding effects of expectation suppression (Summerfield et al., 2008), all stimuli, and each possible pair of stimuli, were presented equally often in a fully randomized order. Participants were required to perform a task incidental to the contrast of interest which involved identifying whether the presented stimuli were familiar or “oddball.” Oddball stimuli, defined as stimuli that did not belong to the training set A to D, were randomly inserted into 10% of trials. Participants were not required to respond if both stimuli on a trial were familiar, but were asked to make a fast button press response if they identified an oddball stimulus. No feedback was given.

The number of trials per block and the number of task blocks varied across experiments (experiment 1: 3 × 25 min task blocks per day, 224 trials per block; experiment 2: 1 × 20 min task block, 208 trials per block; experiment 3: 2 × 20 min task block, 240 trials per block; experiment 4: 2 × 20 min task block per day, 208 trials per block). In both experiments 1 and 4, participants were scanned on a second occasion, 24 hr after the initial scan session.

For experiments 1–3, MRI data were acquired using a 3Tesla Trio MRI scanner (Siemens) with a 32 channel receive-only coil (Siemens) at the Wellcome Trust Centre for Neuroimaging (University College London, UK) and for experiment 4 only, using a 7Tesla Magnetom MRI scanner (Siemens) with 1-channel transmit and a 32-channel phased-array head coil (Nova Medical, USA) at the FMRIB Centre (University of Oxford). Current 7T radio-frequency (RF) coil designs suffer from B1 inhomogeneity effects which were pronounced in the right temporal lobe. To overcome this, we positioned a single barium titanate dielectric pad (4:1 ratio of BaTiO3:D2O, with a relative permittivity of around ∼300, and size 110 × 110 × 5 mm^3^) over the right temporal lobe in all 7T scanning sessions, causing a “hotspot” in the RF distribution at the expense of distal regions (Brink and Webb, 2014, Teeuwisse et al., 2012). During the day 2 scan, the tDCS electrode was situated between the dielectric pad and the head.

For 3T MRI data, an echoplanar imaging (EPI) sequence was used with a 32-channel coil to acquire 20 2.5 mm thick transverse slices with 1 mm gap, in-plane resolution of 3 × 3 mm^2^, repetition time (TR) = 1.4 s, echo time (TE) = 30 ms, flip angle = 90°, and field of view 192 mm. The partial volume covered occipital and temporal cortices and in each session, 850–900 volumes were collected (∼20 min). For each participant, a T1-weighted structural image was acquired to correct for geometric distortions and coregister the EPIs, consisting of 176 1.0 mm axial slices, in plane resolution of 1.0 × 1.0 mm^2^, repetition time = 7.92 s, echo time = 2.48 ms, and field of view = 256 mm. A field map with dual echo-time images was also acquired (TE1 = 10.00 ms, TE2 = 12.46 ms, whole-brain coverage, voxel size 3 × 3 × 2 mm^3^).

For 7T MRI data, an echoplanar imaging (EPI) sequence was used with a 32-channel coil to acquire 24 2.5 mm thick transverse slices with 1 mm gap, in-plane resolution of 2 × 2 mm^2^, repetition time (TR) = 1.4 s, echo time (TE) = 25 ms, flip angle = 60°, and field of view 220 mm. The partial volume covered occipital and temporal cortices and in each session, 850–900 volumes were collected (∼20 min). For each participant, a T1-weighted structural image was acquired to correct for geometric distortions and coregister the EPIs, consisting of 176 0.7 mm axial slices, in-plane resolution of 0.7 × 0.7 mm^2^, repetition time = 2.2 s, echo time = 2.96 ms, and field of view = 224 mm. A field map with dual echo-time images was also acquired (TE1 = 4.08 ms, TE2 = 5.1 ms, whole-brain coverage, voxel size 2 × 2 × 2 mm^3^).

### MRS

On day 2 of experiment 4, MRS was acquired from 21 of the 25 participants. B0 shimming was performed in a two-step process. First, GRE-SHIM (field of view, 384 × 384 mm^2^; TR = 600 ms; TE1/2 = 2.04/4.08 ms; slice thickness 4 mm; flip angle 15°; slices 64; scan time 45 s) was used to determine the optimal first- and second-order shim currents (Shah et al., 2009). The second step involved only fine adjustment of first-order shims using FASTMAP (Gruetter and Tkác, 2000). The modified semi-LASER sequence, previously shown to have minimal chemical shift displacement error (CSDE), was used with TE = 36 ms, TR = 5–6 s to acquire MRS measurements in a 2 × 2 × 2 cm^3^ volume of interest (VOI), positioned next to the tDCS electrode (Figure S1C) (van de Bank et al., 2015, Oz and Tkáč, 2011).

For each MRS measurement between 96 and 128, scan averages were collected, giving a total acquisition time of around 10 min. Three measurements were acquired for each participant, before and during tDCS, and again after the second task block (Figure 3C).

Metabolites were quantified using LCModel (see Supplemental Experimental Procedures; see also Figures S1A and S1B) (Provencher, 1993, Provencher, 2001). Relative to baseline concentrations, the change in GABA (Figures 3E and 5A), glutamate (Figure 5B), and other metabolite concentrations was estimated both during tDCS and post-task using a two-tailed paired t test where the direction of the effect was unknown and a one-tailed paired t test in instances where the direction of the effect was predicted from previous data (i.e., for GABA).

### tDCS

On day 2 of experiment 4, a DC-Stimulator (Eldith) delivered a 1 mA current to the brain while the participants were inside the 7T MRI scanner. To allow for tDCS to be delivered inside the 7T scanner, two 5 × 7 cm MRI compatible electrodes (Easycap) were fitted with 5 kOhm resisters to minimize the risk of heating or eddy current induction. Using high-chloride EEG electrode gel (Easycap) as a conducting paste, the anodal electrode was placed on the scalp above the region of right temporal cortex previously identified as encoding the association between paired shapes (Figure 3D), approximately at the 10–20 T6 node location. The cathodal electrode was placed over the contralateral supraorbital ridge. A cod-liver oil capsule was taped to the center of the anodal electrode to make the electrode MR-visible and allow for its location to be mapped onto the anatomical brain surface (Figure S1C). The impedance of tDCS was checked prior to the participant entering the scanner and again once the participant was lying inside the bore of the magnet with extension leads connected to the stimulator. tDCS involved a 10 s ramp up of the current, which was then held at 1 mA current for a total of 20 min, before being ramped down over 10 s. tDCS commenced after the first MRS measurement acquisition (baseline), 10 min prior to the start of the second fMRI task session (see Figure 3C).

### Postscan Behavioral Task

On day 2 of experiment 4, immediately after participants exited the scanner they were given a surprise memory test (see Figure 3C). This involved the three alternative forced choice design used in the behavioral training, but in the absence of feedback (mean number of trials, 22.7).

### fMRI Data Analysis

All MRI datasets were preprocessed using SPM (http://www.fil.ion.ucl.ac.uk/spm/). Images were corrected for signal bias, realigned to the first volume, corrected for distortion using field maps, normalized to a standard EPI template and smoothed using an 8 mm full-width at half maximum Gaussian kernel.

For each participant and for each scanning block, fMRI data was analyzed in an event-related manner using a general linear model (GLM) in SPM. Explanatory variables used a delta function to indicate the onset of a trial and were then convolved with the hemodynamic response function. Explanatory variables were included for trials with associated stimuli (e.g., A followed by B, or C followed by D), unrelated stimuli (e.g., A followed by C or B followed by D), and repeated stimuli (e.g., A followed by A). In experiment 1, an additional explanatory variable was included to account for trials with stimulus E. In experiment 2, the “unrelated” explanatory variable was divided in two (i.e., C and D trials divided) to allow for an orthogonal test of cross-stimulus adaptation. In all experiments, an additional six scan-to-scan motion parameters produced during realignment were included in the GLM as additional nuisance explanatory variables to account for motion-related artifacts.

To measure cross-stimulus adaptation the contrast of interest involved comparing the BOLD response to associated stimuli with that of unrelated stimuli (“unrelated” minus “associated”). Notably, this contrast controlled for attention-dependent differences in expectation suppression across sessions (Larsson and Smith, 2012). The contrast images of all participants were entered into a second-level random effects analysis. To test for cross-stimulus adaptation in an unbiased fashion, parameter estimates obtained from the GLM were extracted from an independent ROI (see Supplemental Experimental Procedures for ROI definitions), and contrasted using a two-tailed t test where the direction of the effect was unknown, and a one-tailed t test in instances where the direction of the effect was predicted from previous data. Two-tailed paired t tests were used to assess differences across sessions.

### Network Modeling

See Supplemental Information for experimental procedures concerning the network modeling.

## Author Contributions

All of the authors contributed to the design of the study, preparation of the manuscript, and design of the figures. H.C.B. acquired the data with U.E.E, T.R.M, J.O., S.C., and T.E.J.B. Data were analyzed by H.C.B with U.E.E., S.J., and T.E.J.B.; T.P.V. generated all simulations.

## Supplemental Information

Document S1. Figures S1–S6, Tables S1 and S2, and Supplemental Experimental Procedures

Document S2. Article plus Supplemental Information
