# Supplementary figures and images for: Unmasking Latent Inhibitory Connections in Human Cortex to Reveal Dormant Cortical Memories
Source: Neuron. 2016 Apr 6;90(1):191–203. doi: 10.1016/j.neuron.2016.02.031 (PMC4826438; doi:10.1016/j.neuron.2016.02.031)

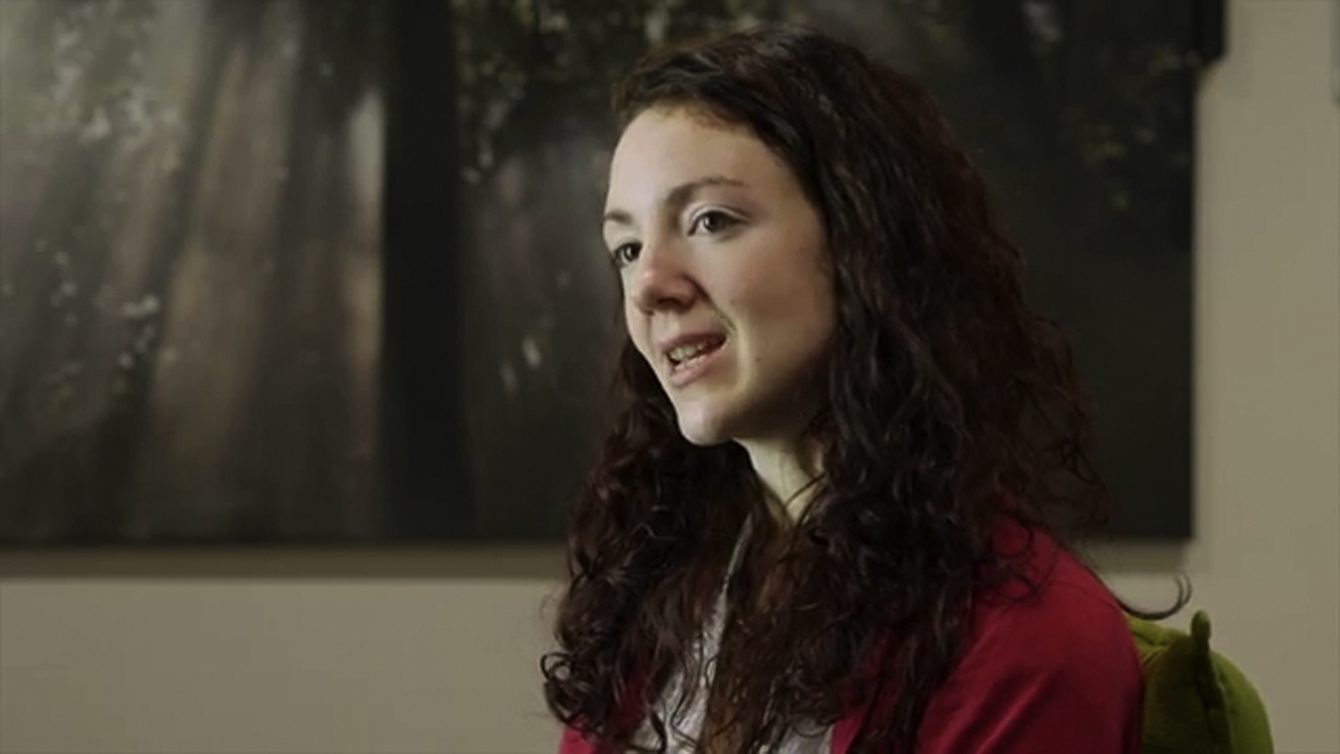

Supplement: Supplementary file 1 [file mmc3.jpg]
