# Supplementary material for: Unmasking Latent Inhibitory Connections in Human Cortex to Reveal Dormant Cortical Memories
Source: Neuron. 2016 Apr 6;90(1):191–203. doi: 10.1016/j.neuron.2016.02.031 (PMC4826438; doi:10.1016/j.neuron.2016.02.031)
Supplement: Document S1. Figures S1–S6, Tables S1 and S2, and Supplemental Experimental Procedures [file mmc1.pdf]

Neuron, Volume *90*

Supplemental Information

Unmasking Latent Inhibitory Connections

in Human Cortex to Reveal Dormant Cortical Memories

H.C. Barron, T.P. Vogels, U.E. Emir, T.R. Makin, J. O'Shea, S. Clare, S. Jbabdi, R.J. Dolan, and T.E.J. Behrens

Supplemental Figure S1

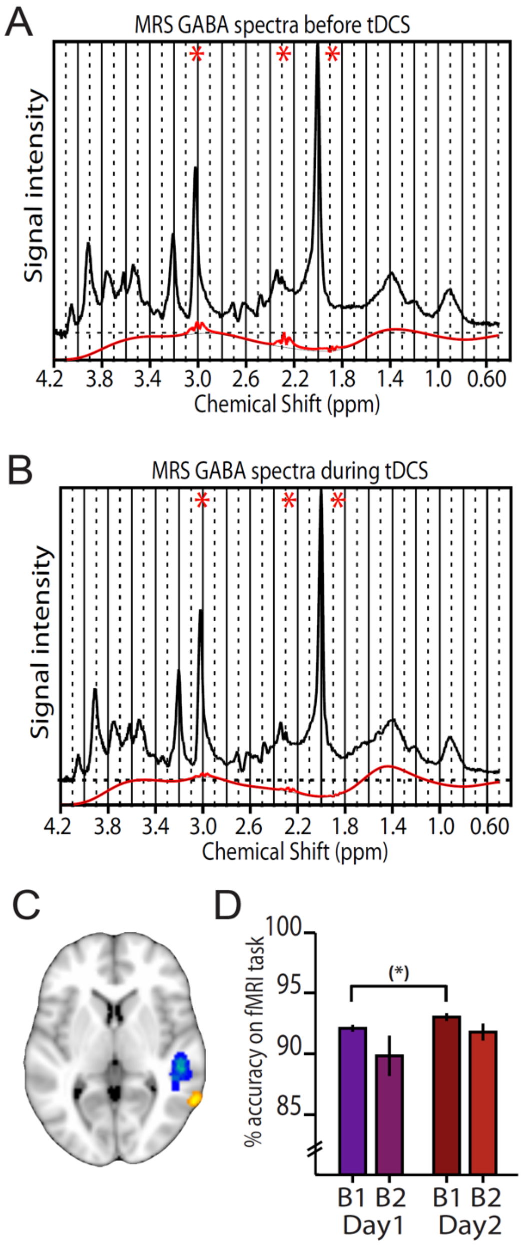

Figure S1 (related to Figure 3) | MRS spectra, MRS voxel location and behavioural analyses
A-B Example MRS spectra for a single participant after 10mins acquisition. In black is the LCModel fit. In red is the GABA spectra estimated by LCModel. The three peaks contributing to the GABA spectra are indicated with stars. **A** MRS spectra at baseline, before tDCS. **B** MRS spectra during tDCS, where the signal intensity of the GABA peaks is reduced relative to baseline shown in ***A***. **C** Average tDCS electrode location (orange) and average MRS voxel location (blue). **D** Percentage of trials with the correct behavioural response made during the fMRI task (mean ± SEM). There was near significant improvement in performance from day 1 to day 2 (Day1 B1 vs Day2 B1, p=0.054).

Supplemental Figure S2

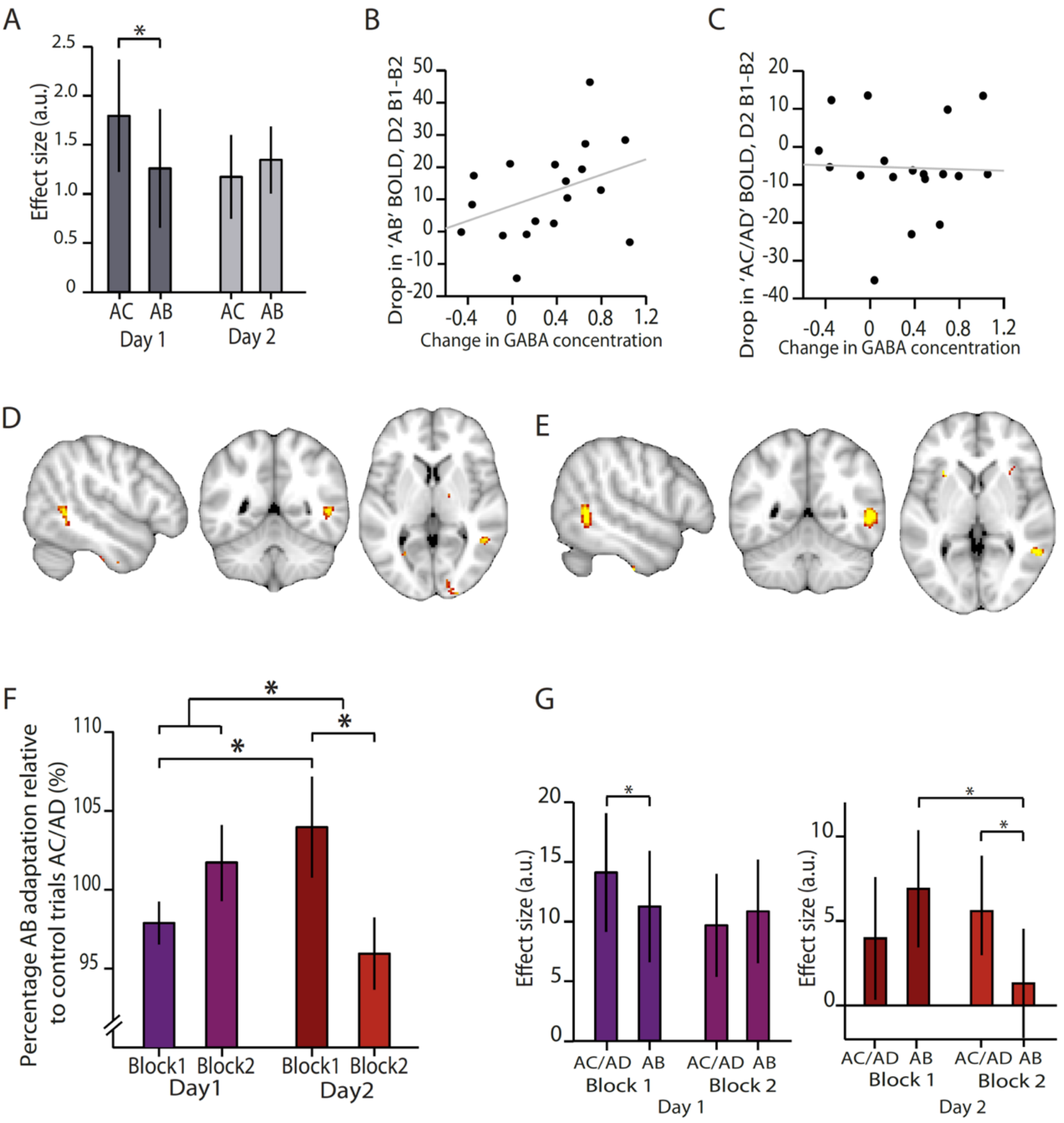

Figure S2 (related to Figures 2 and 3) | Cross-stimulus adaptation: decomposition of data analyses
A One set of pilot participants were scanned on a second occasion, 24 hours after the initial scan, and showed significant decrease in cross-stimulus adaptation ('AC' – 'AB') on day 2 compared to day 1 (Fig. 2B). Decomposing this effect here, the BOLD response to consecutive presentation of two un-associated stimuli ('AC') and to consecutive presentation of two associated stimuli ('AB') can be seen for both days (mean ± SEM). **B** Consistent with the relationship between GABA and cross-stimulus adaptation ('AC'/'AD' – 'AB') shown in Fig. 3G, the change in GABA concentration before vs. during tDCS showed a correlational trend with the change in the BOLD response to consecutive presentation of two associated stimuli ('AB') from Day2 (D2) Block1 (B1) to Block2 (B2) (change in GABA vs. drop in 'AB': r_{17}= -0.379, p=0.121). **C** Consistent with the relationship between GABA and cross-stimulus adaptation ('AC'/'AD' – 'AB') shown in Fig. 3G, the change in GABA concentration before vs. during tDCS did not show correlation with the change in BOLD response to consecutive presentation of two un-associated stimuli ('AC'/'AD') from Day2 (D2) Block1 (B1) to Block2 (B2) (change in GABA vs. drop in 'AC'/'AD': r_{17}= -0.032, p=0.898). **D** As shown in Fig. 3F, an anterior region of the lateral occipital complex showed significant interaction between the cross-stimulus adaptation effect measured across blocks and days, within the ROI defined from the mean tDCS electrode location (Fig. 3D). This interaction effect is observed here on a whole brain map, thresholded at P <0.01 uncorrected to aid visualisation. **E** As shown in Fig. 3F, an anterior region of the lateral occipital complex showed a significant

increase in cross-stimulus adaptation following application of tDCS (day2, block2-block1), within the ROI defined from the mean tDCS electrode location (Fig. 3D). This increase in cross-stimulus adaptation is observed here on a whole brain map, thresholded at P <0.01 uncorrected to aid visualisation. **F** To complement the analysis shown in Fig. 3F, the BOLD response to paired stimuli ('AB') in experiment 4 is shown here as a percentage proportion of the BOLD response to unpaired stimuli ('AC'/'AD') (mean ± SEM). As shown in Fig. 3F, there was significantly greater adaptation in Block1-Day1 compared to Block1-Day2 (t_{20}=1.46, p=0.040), a significant increase in adaptation from Block1-Day2 to Block2-Day2 following application of tDCS (t_{20}=2.53, p=0.005), and a significant interaction in adaptation across blocks and days (t_{20}=2.44, p=0.006). This shows that the significant change in cross-stimulus adaptation reported in Fig. 3F cannot be attributed to basic changes in cortical excitability. **G** This panel complements Fig. 3F by showing separate parameter estimates for the response to paired stimuli ('AB') and unpaired stimuli ('AC'/'AD') for each block across both days in experiment 4 (mean ± SEM). These plots show that there was significant adaptation in Day1-Block1 (t_{20}=1.79, p=0.044) and Day2-Block2 (t_{20}=1.97, p=0.032), and a significant decrease in the BOLD response to paired stimuli from block1 to block2 on day2 (t_{20}=1.96, p=0.032). This again suggests that cross-stimulus adaptation effects reported in Fig. 3F cannot be attributed to basic changes in cortical excitability. Notably, however, in this decomposition of the data differences in attention-dependent expectation suppression across blocks are not controlled for.

Supplemental Figure S3

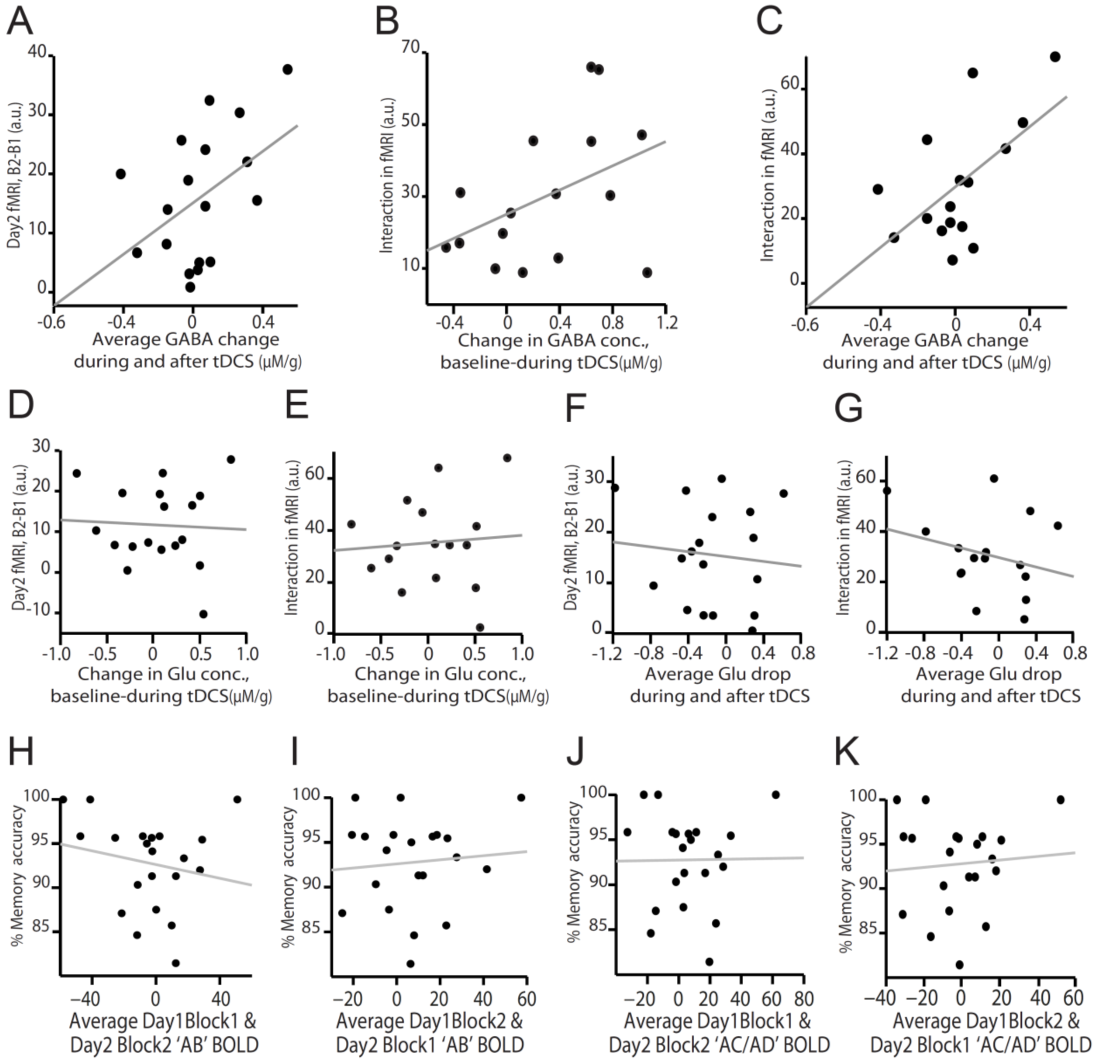

Figure S3 (related to Figures 3 and 4) | Relationships between cross-stimulus adaptation and the change in metabolite concentrations with tDCS, and between cross-stimulus adaptation and memory accuracy
Panels **A – G** complement Fig. 3G, **H – I** complement Fig. 4B and **J – K** complement Fig. 4C. **A** The change in GABA concentration before vs. the average concentration during tDCS and post task showed near significant correlation with the change in cross-stimulus adaptation from Day2 Block 1 to Day2 Block 2 (with effects due to glutamate removed, r_{17}=0.456, p=0.057). **B** The change in GABA concentration before vs. during tDCS showed near significant correlation with the interaction in cross-stimulus adaptation between Day2 and Day1 (with effects due to glutamate removed, r_{15}=0.430, p=0.096). **C** The change in GABA concentration before vs. the average concentration during tDCS and post task showed significant correlation with the interaction in cross-stimulus adaptation between Day2 and Day1 (with effects due to glutamate removed, r_{15}=0.588, p=0.017). **D** The change in glutamate concentration before vs. during tDCS did not show correlation with the change in cross-stimulus adaptation from Day2 Block 1 to Day2 Block 2 (with effects due to GABA removed, r_{17}=-0.053, p=0.836). **E** The change in glutamate concentration before vs. during tDCS did not show correlation with the interaction in cross-stimulus adaptation between Day2 and Day1 (with effects due to GABA removed, r_{15}=-0.079, p=0.773). **F** The change in glutamate concentration before vs. the average concentration during tDCS and post task did not show correlation with the change in cross-stimulus adaptation from Day2 Block 1 to Day2 Block 2 (with effects due to GABA removed, r_{17}=-0.107, p=0.673). **G** The change in glutamate concentration before vs. the average concentration during tDCS and post task did not correlate with the interaction in cross-stimulus adaptation between Day2 and Day1 (with effects due to GABA removed, r_{15}=-0.279, p=0.296). **H** While we observed a positive relationship between memory accuracy and cross-stimulus adaptation in Fig. 4B, memory accuracy did not show a significant negative correlation with the BOLD response to consecutive

presentation of two associated stimuli (‘AB’) during periods of EI imbalance (Day1 Block1 and Day2 Block2) (r_{20}=-0.196, p=0.395). **I** Consistent with Fig. 4C, memory accuracy did not show correlation with the BOLD response to consecutive presentation of two associated stimuli (‘AB’) during periods of EI balance (Day1 Block2 and Day2 Block1) (r_{20}= -0.091, p=0.694). **J** Memory accuracy did not show a significant correlation with the BOLD response to consecutive presentation of two unrelated stimuli (‘AC’ / ‘AD’) during periods of EI imbalance (Day1 Block1 and Day2 Block2) (r_{20}= -0.012, p=0.958). **K** Memory accuracy did not show a significant correlation with the BOLD response to consecutive presentation of two unrelated stimuli (‘AC’ / ‘AD’) during periods of EI balance (Day1 Block2 and Day2 Block1) (r_{20}= -0.083, p=0.720).

Supplemental Figure S4

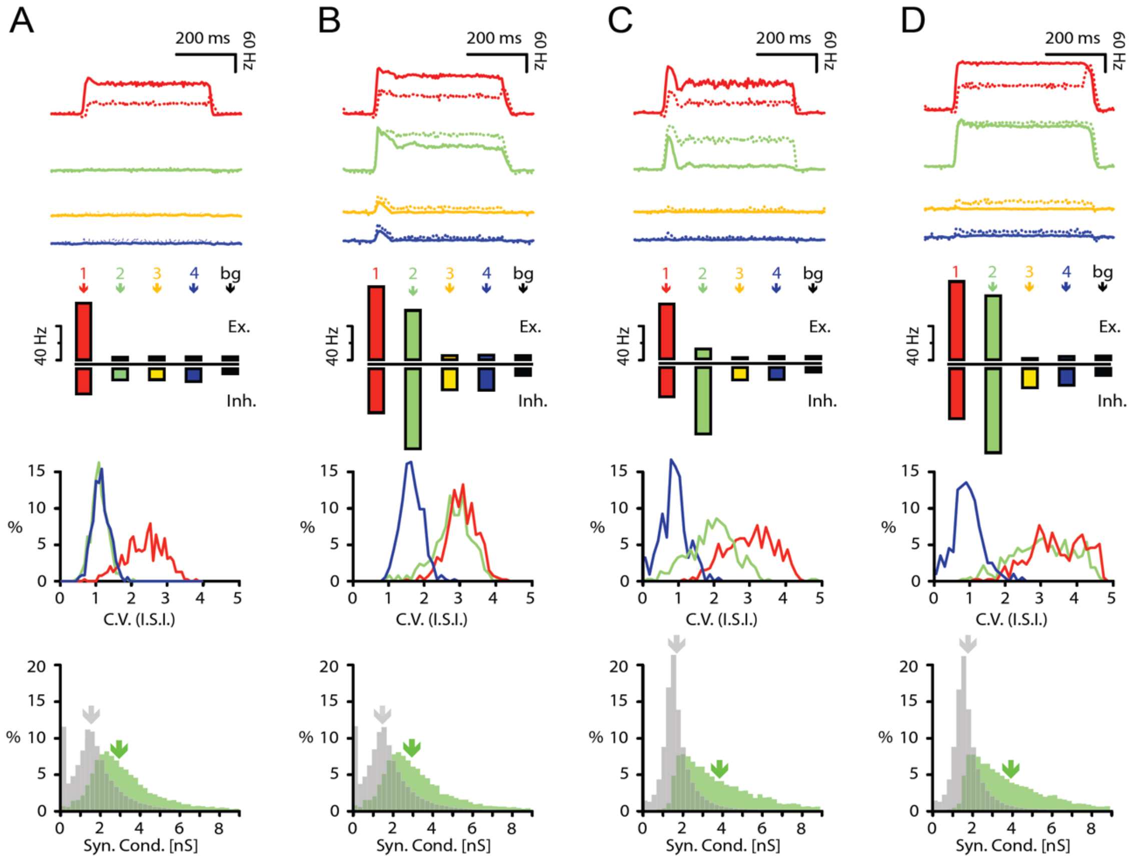

Figure S4 (related to Figure 6) | Additional network model data

Each panel **A – D** provides additional information for the identically named panels of Fig. 6. The first row shows, in solid lines, the same average firing rates of the excitatory neurons averaged over 5 trials as in the penultimate row of Fig. 6. Additionally, the dotted lines show the firing rates of the local inhibitory neurons. The second row shows the average firing rate over all excitatory and local inhibitory neurons for each assembly, and for the background neurons (bg) over the 1s duration of a stimulus trial. The third row shows the distributions of coefficient of variation (C.V.) of interspike intervals (I.S.I.) of all neurons in the red and green assemblies, as well as all non-assembly neurons, shown in red, green and black, respectively. The fourth row shows the distributions of strengths of local inhibitory synapses originating from local inhibitory neurons in the green assembly, and from non-assembly local inhibitory neurons, shown in green and grey respectively. The arrows note the average value of each distribution. **A** In the initial, balanced state, the upper left (red) cell assembly is activated by a targeted decrease of inhibitory activity in the assembly. **B** When excitatory connections between associated cell-assemblies were selectively enhanced, the activation of the same assembly co-activates the associated green cell-assembly. **C** After inhibitory plasticity balanced the surplus excitation, stimulation results in only brief co-activation of the associated green cell assembly before inhibitory activity silences the paired assembly. **D** Reducing the efficacy of all inhibitory synapses in the balanced network, restored co-activation of the associated cell assembly (green) in response to driving the red cell assembly.

Supplemental Figure S5

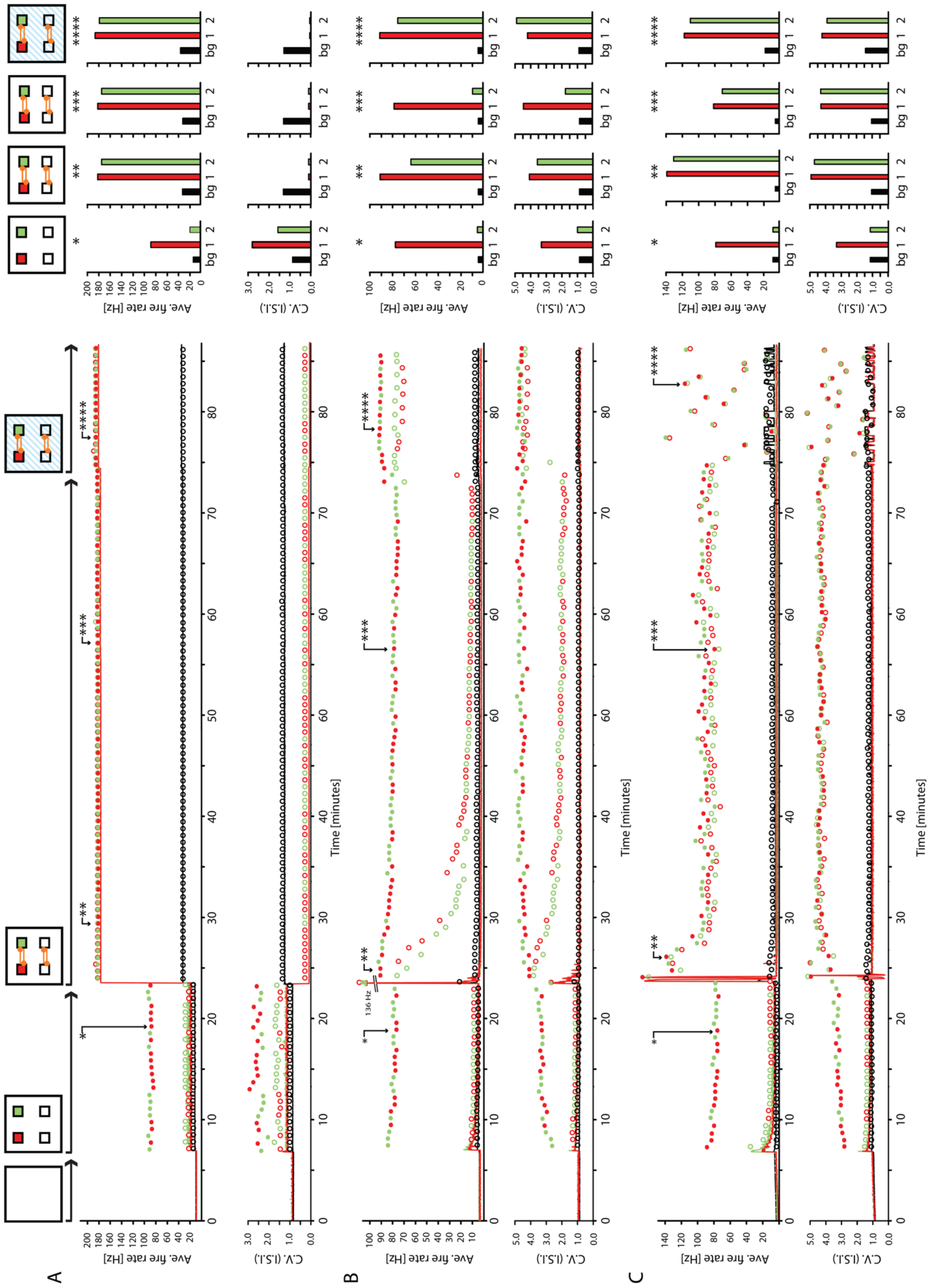

Figure S5 (related to Figure 6) | Performance comparison of the network with an alternative model using homeostatic scaling to rebalance the network.

Each row **A – C** shows the average firing rates (upper panel) and coefficient of variation of interspike intervals (C.V.(I.S.I.), lower panels) of the complete simulation of all stages of the protocol Fig. 6A-D in 80 minutes, with accordingly adjusted learning rate η. Solid lines show the average firing rate / C.V.(I.S.I.) of the red and green cell assemblies over 2 seconds, and the activity / C.V.(I.S.I.) of all background neurons is plotted in black in the upper and lower panels respectively. Circles show the average firing rate / C.V.(I.S.I.) of red and green assembly neurons when they are stimulated (solid circles) or when the other assembly is stimulated (open circles), at 40s intervals. Open black circles show the firing rates / C.V.(I.S.I.) of un-stimulated background neurons during stimulations. The simulation begins with a naïve network without assembly structure, firing at 5 Hz. At t = 7 mins, four cell assemblies are introduced by strengthening all excitatory weights within the assembly. At t = 23.5 minutes, 'associative' excitatory synapses *between* the red and green, and the blue and yellow (not shown) cell assemblies are introduced. At t = 74 minutes, all inhibitory synapses within the network are reduced in strength by 15%. **A** The effect of synaptic changes as described above on firing rate and C.V.(I.S.I.), without any compensatory mechanism. Network activity more destabilizes with each consecutive manipulation. **B** Firing rate and C.V.(I.S.I.) for the same protocol when inhibitory synaptic plasticity is active. Network activity is stable and assemblies can be individually activated after inhibitory synaptic plasticity reaches steady state. **C** Firing rate and C.V.(I.S.I.) for the same protocol when homeostatic, activity dependent plasticity of excitatory synapses is active. Network activity is stable, but associated assemblies can henceforth never individually activated again.

Supplemental Figure S6

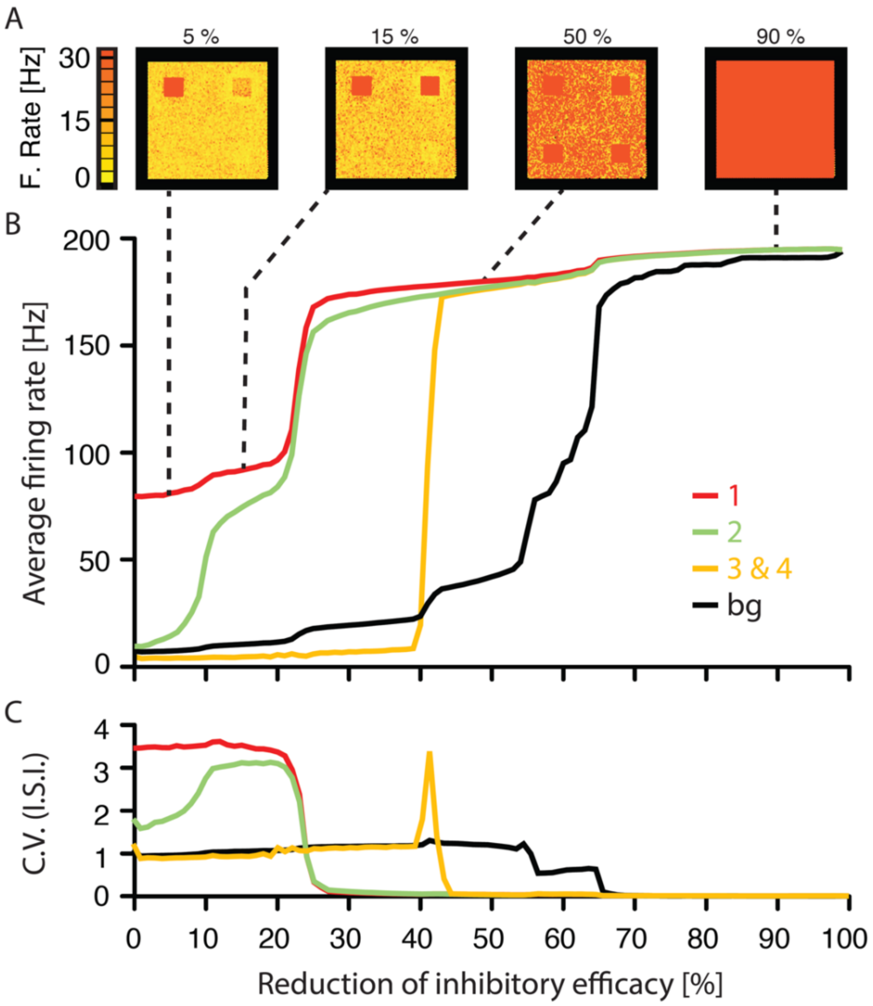

Figure S6 (related to Figure 6) | Network response to different levels of simulated GABAergic reduction during recall of a cell assembly

A Snapshots of all network neurons' average activity during recall over 1 second, plotted on a 144x144 raster. Inhibitory synaptic efficacy was reduced by 5%, 15%, 50%, and 90% from left to right. **B** The network was initially in the balanced state, as shown in Fig. 6C, before the efficacy of inhibitory connections was incrementally reduced from 0% to 100%. During this incremental reduction in inhibition, the average population activity over 20 x 500ms recall episodes of the red cell assembly was assessed. The average response of each cell assembly is shown separately (red, green, blue and yellow) along with the average response of the background assembly (black). Reliable co-activation of the associated cell assemblies (green co-activation in response to recall of red) can be observed when the percent reduction in efficacy of inhibitory connections falls within the approximate range 10%-40%. Further reduction (>~40%) causes spontaneous recall of un-associated memories (yellow and blue cell assemblies), before the entire network transitions into a chaotic state (>~60% reduction). Dashed lines indicate reduction levels in the 4 snapshots shown in *A*. **C** Average coefficient of variation of the inter spike intervals during the same recall episodes, grouped as in *B*.

Supplemental Tables S1-S2:

Paired stimulus	n	Scanner strength	Relevant figure	Cross-stimulus adaptation location	Adaptation contrast	Shapes presented during scan task	Contrast for orthogonal ROI	t-test for adaptation
coloured shapes	9	3T	Fig. 1D,1E,1F, 1J, 2B	occipital & temporal cortices	AC - AB	A,B,C,E	AE- AA	t=1.96, P=0.043
rotationally invariant shapes	8	3T	Fig. 1G, 1H, 1I, 1J	anterior LOC	AC - AB	A,B,C,D	AD- AA	t=2.41, P=0.024
stimulus with food reward	10	3T	Fig. 1J	lateral OFC	AC or AD - AB	A,B,C,D	Taken from Klein-Flügge*, Barron* et al., Journal of Neurosci. 2013. Fig. 3a.	t=2.11, P=0.032
imagined foods	19	3T	Fig. 1J, Barron et al., Nature Neurosci. 2013	medial PFC	AC or AD - AB	A,B,C,D	Taken from Barron et al., Nature Neurosci. 2013, Fig. 4a.	t=4.24, P=0.014
rotationally invariant shapes	21	7T	Fig. 3B, 3F, 3G	anterior LOC	AC or AD - AB	A,B,C,D	Mean tDCS electrode location	t=1.79, P=0.044

Table S1 (related to Figures 1-3) | Measuring cortical associations in humans using cross-stimulus adaptation

Experimental and analytical details for the five different experiments used to measure cortical associations via cross-stimulus adaptation.

MRS measurement	SNR	FWHM	CRLB(%) GABA	CRLB(%) Glutamate
Before tDCS	46.1±9.6	10.6±1.7	21.7±6.0	3.8±0.7
During tDCS	46.3±6.8	10.2±9.6	23.2±6.5	3.8±0.7
Post task	46.9±8.6	10.6±9.6	21.6±7.1	3.8±0.7

Table S2 (related to Figures 3 and 5) | MR Spectral quality table.

Data averaged across participants to allow for comparison across the three MRS measurements. The table shows the following: Signal to noise ratio (SNR) ± standard deviation (SD), Full width at half maximum (FWHM) ±SD, and Cramer Rao Lower Bounds (CRLB) ±SD for GABA and glutamate.

Supplemental Experimental Procedures

MRS- LCModel metabolite quantification

Metabolites were quantified using LCModel (for example spectra: Fig. S1A, S1B) (Provencher, 1993, 2001). The model spectra of alanine (Ala), aspartate (Asp), ascorbate/vitamin C (Asc), glycerophosphocholine (GPC), phosphocholine (PCho), creatine (Cr), phosphocreatine (PCr), GABA, glucose (Glc), glutamine (Gln), glutamate (Glu), glutathione (GSH), myo-inositol (myo-Ins), Lactate, N-acetylaspartate (NAA), N-acetylaspartylglutamate (NAAG), phosphoethanolamine (PE), scyllo-inositol (scyllo-Ins) and taurine (Tau) were generated based on previously reported chemical shifts and coupling constants by VeSPA Project (Versatile Simulation, Pulses and Analysis) (Govindaraju et al., 2000; Soher et al., 2011; Tkac et al., 2008).

The unsuppressed water signal acquired from the VOI was used to remove residual eddy current effects and to reconstruct the phased array spectra (Natt et al., 2005). Single scan spectra summed from 32 channels were corrected for frequency and phase variations induced by subject motion, and then summed. LCModel analysis was performed on all spectra within the chemical shift range 0.5 to 4.2 ppm (Provencher, 1993). Reliable LCModel fits were achieved in 18 of the 21 participants and metabolite concentration obtained relative to the unsuppressed water spectrum acquired from the same VOI. The full width at half maximum (FWHM) and SNR determined by LCModel were not different across conditions (Table S2). Only metabolites quantified with Cramér–Rao lower bound (CRLB) $\leq$ 50% were included in the final neurochemical profile, which in this instance corresponded to all measured metabolites (Bednařík et al., 2015). Given the consistency in SNR and FWHM across all conditions, higher CRLBs observed during tDCS stimulation (Table S2) can be attributed to the reduction in GABA signal in the voxel during tDCS stimulation (Emir et al., 2012).

fMRI data analysis- ROI specification

To assess cross-stimulus adaptation in experiments 1 and 2 (Fig. 1F, 1I, 1J, 2B), ROIs were defined using an orthogonal contrast to the contrast of interest (see Table S1). This orthogonal contrast was first thresholded to give clusters. We then searched across each hemisphere to find the voxel with the highest t-value. The cluster which contained this voxel was then selected as the ROI. For early visual responses (Fig. 1F, 2B), we found one cluster in each hemisphere, giving bilateral ROIs. For the LOC response (Fig. 1I) we only searched in the right hemisphere since subsequent application of tDCS could only be applied to one hemisphere (experiment 4) and LOC notably shows a right lateralized response to object matching (Large et al., 2007). To assess cross-stimulus adaptation in experiment 3 (lOFC in Fig. 1J), an independent ROI was taken from a previous publication (see Table S1) (Klein-Flügge et al., 2013). To assess cross-stimulus adaptation in experiment 4 (Fig. 3F), an unbiased ROI was defined from the peak location of the tDCS electrode, with the x-coordinate defined using the average of three peaks from pilot experiment 2 (Fig. 3D). In each case, a measure of cross-stimulus adaptation was obtained by comparing the parameter estimates for 'unrelated' and 'associated' conditions.

In experiment 4, the relationship between cross-stimulus adaptation and the change in GABA concentration (Fig. 3G), and the relationship between cross-stimulus adaptation and memory performance (Fig. 4B, 4C), were assessed using individual specific ROIs, defined from the peak interaction effect (Fig. 3F). For each individual, a cluster was first selected by thresholding the interaction contrast at $p<0.01$ uncorrected and taking the cluster closest to the average tDCS electrode location (Fig. 3D). The peak voxel of this cluster was then selected for the ROI, and smoothed using a 5-mm full-width at half maximum Gaussian kernel to give a 4-voxel diameter sphere. Parameter estimates were then extracted from these individual-specific ROIs and used to perform correlations with memory performance (Fig. 4B and 4C), and partial correlations between changes in cross-stimulus adaptation and GABA concentration following tDCS, with effects due to glutamate removed (Fig. 3G; see also Fig. S3A-C). Similarly, partial correlations between changes in cross-stimulus adaptation (or raw BOLD) and glutamate concentration following tDCS were calculated, after effects due to GABA were removed (Fig. S3D-G).

To assess changes in the raw BOLD response (Fig. 5D, 5E) and avoid confounding the analysis with adaptation effects, parameter estimates were extracted from an ROI defined from the average BOLD response to pairs of unrelated stimuli ('A' followed by 'C' etc.) across all task blocks (Fig. 5C). This ROI was defined in the same way as early visual ROIs used in experiment 1 and 2 above, by taking bilateral clusters which had the highest t-value in each hemisphere of the thresholded orthogonal contrast. This ROI corresponded to the brain region that maximally responded to task stimuli in a functionally non-selective manner. Within this brain region, the raw BOLD response to pairs of unrelated, non-adapting, stimuli ('A' followed by 'C' etc.) was assessed before and

after application of tDCS using a paired *t* test. The relationship between this change in BOLD response and the change in metabolite concentration was considered using multiple regression. The change in concentration of GABA and glutamate were included as explanatory variables. A set of contrasts were used to assess the main effect of each metabolite and the interaction between metabolites.

Network Modelling

For the network modelling we combined two previous studies and followed their methods closely (Vogels and Abbott, 2009; Vogels et al., 2011). We used standard leaky integrate-and-fire (LIF) neurons that were characterized by a time constant, $\tau = 20$ ms, and a resting membrane potential, V_{rest} = -60 mV. Whenever the membrane voltage crossed a spiking threshold of -50 mV, an action potential was generated and the membrane voltage set back to the resting potential, where it remained clamped for a 5 ms refractory period. To set the scale for currents and conductances in the model, we used a membrane resistance of 100 MΩ (g_{leak} = 10 nS).

Synapses onto each neuron were modelled as conductances, so the sub-threshold membrane voltage obeyed

$$\tau \frac{dV}{dt} = (V_{\text{rest}} - V) + (g_{\text{ex}}(E_{\text{ex}} - V) + g_{\text{inh}}(E_{\text{inh}} - V) + I_b)/g_{\text{leak}}.$$

Reversal potentials were E_{ex} = 0 mV and E_{inh} = -80 mV. The synaptic conductances g_{ex}, g_{inh} were expressed in units of the resting membrane conductance. When the neuron received a presynaptic action potential, the appropriate postsynaptic variable was increased, $g_{\text{ex}} \rightarrow g_{\text{ex}} + \Delta g_{\text{ex}}$ for an excitatory spike, and $g_{inh} \rightarrow g_{inh} + \Delta g_{inh}^{local}$ or $g_{inh} \rightarrow g_{inh} + \Delta g_{inh}^{global}$ for inhibitory spikes from local or global inhibitory neurons, respectively. Otherwise, these parameters obey the equations

with synaptic time constants τ_{ex} = 5 ms and τ_{inh} = 10 ms. The conductance of each synapse was constructed

$$\tau_{\text{ex}} \frac{dg_{\text{ex}}}{dt} = -g_{\text{ex}} \text{ and } \tau_{\text{inh}} \frac{dg_{\text{inh}}}{dt} = -g_{\text{inh}}$$

such that $\Delta g_{ij} = \bar{g} W_{ij}$ where $\bar{g}$ is a constant (except where noted as postsynaptic factor $\bar{g}_i$ for simulations of Fig. S5C, see below). W_{ij} could be plastic or fixed, depending on the identity of the synapse (see below). I_b = 300 pA corresponded to a constant background current used to maintain network activity. The integration time step for our simulations was 0.1 ms.

Except for synapses within or between assemblies, all synapses from excitatory or global inhibitory neurons had the same strength. They were adjusted to allow asynchronous background activity within the network, i.e. the postsynaptic conductances were set such that with $W_{ij} = 1, \Delta g_{\text{ex}} = 0.8$ nS. and $\Delta g_{\text{inh}}^{\text{global}} = 7.5$ nS, corresponded to 0.5 mV EPSPs and -1.1 mV IPSPs respectively, as obtained from spike triggered averages in the active network. Local inhibitory synapses were initially set to $\Delta g_{\text{inh}}^{\text{local}} = 1.5$ nS (-0.4 mV IPSPs), but their strength could be changed by the synaptic plasticity rule described below. Under these conditions the network was intitially sufficiently balanced to prevent instable network dynamics.

Following Vogels et al. (2011), we implemented a synaptic plasticity rule that potentiated synapses upon coincident pre- and postsynaptic activity within a coincidence time window τ_{STDP}. Additionally, sole presynaptic spikes led to a reduction of synaptic efficacy. For the sake of simplicity, and in accordance with the experimentally still ambiguous situation (Vogels et al., 2013), we utilized a symmetric spike-timing dependent learning rule between a presynaptic neuron j and a postsynaptic neuron i. Potentiation occurred as a function of $\Delta t = |t_i^f - t_j^f|$ (in which t_i^f and t_j^f denote the time of a pre- and postsynaptic spike respectively). Depression occurred for each presynaptic spike by a fixed amount α. This spike-timing dependent plasticity (STDP) rule was implemented for local inhibitory synapses projecting onto excitatory cells. In order to calculate the changes to each W_{ij}, a synaptic memory trace x_i was assigned to each neuron. x_i increased with each spike $x_i \rightarrow x_i + 1$

and otherwise decayed, following $\tau_{STDP} \frac{dx_i}{dt} = -x_i$ with time constant τ_{STDP} = 20 ms. The synaptic weight W_{ij} from neuron j to neuron i was updated for every pre- or postsynaptic event such that:

$W_{ij} \rightarrow W_{ij} + \eta\,(x_i - \alpha)$ for presynaptic spikes at time t_j^f and
$W_{ij} \rightarrow W_{ij} + \eta\,x_j$ for postsynaptic spikes at time t_i^f

where η is the learning rate, $\alpha = 2 \times \rho_0 \times \tau_{STDP}$ the depression factor, and ρ_0 a constant parameter with units 1/time (Vogels et al., 2011). For the simulations shown here, we set $\eta = 10^{-5}$ and $\alpha = 0.24$ (ρ_0 = 6 Hz).

For a single set of simulations (Fig. S5C) we turned off inhibtory synaptic plasticity ($\eta = 0$) and instead used a form of activity dependent (homeostatic) scaling to stabilize network activity scaling (Rossum et al., 2000). The mechanism adjusted all excitatory presynaptic weights of the same postsynaptic cell by changing the common factor $\bar{g}_i$, such that

$$\frac{d\bar{g}_i}{dt} = \beta \bar{g}_i[\kappa - a]$$

where κ=6 Hz is the desired postsynaptic activity, a is a slow-varying sensor that measures the average postsynaptic activity and $\beta = 10^{-3}$ is a constant that determines the effective speed of the scaling (Rossum et al., 2000). For computational efficiency and network stability we updated the weights every 5 ms, and a could be integrated over as little as 5ms without general loss of stability.

As in Vogels & Abbott (2009), the network we studied was composed of 20,164 LIF neurons, laid out on a 142 x 142 grid. Neurons were either excitatory or inhibitory. The ratio of inhibitory neurons was roughly one in four, but the geometric organization of neurons on the grid constrained the final numbers to 15,123 excitatory cells and 5,041 inhibitory cells. Inhibitory neurons were divided into two groups of 3,361 and 1,680 neurons that differed in their connectivity pattern. All excitatory neurons and 65% of the inhibitory neurons had a random connectivity of 2% to the rest of the network . The 1,680 inhibitory neurons of the second group each targeted 40% of their 500 closest neighbors and thus acted locally. To avoid boundary effects, the network had the topology of a torus. Other network parameters were chosen in keeping with both general properties of cortical circuits and previous work (Rossum et al., 2002; Vogels and Abbott, 2005, 2009).

In addition to the general architecture, we introduced specific patterns into the weight matrix by defining four groups of 22×23 = 506 neurons as Hebbian assemblies. We strengthened all existing excitatory connections between the neurons within each assembly by a factor of ten. For simplicity, only the local inhibitory to excitatory connections in the network were plastic. Further, we assumed that the structure of the connectivity matrix remained fixed after the network had been initialized. This restricted inhibitory plasticity operations to existing connections. Note however that the weight of an existing connection W_{ij} could decay to zero.

To simulate the display of a stimulus, the balance between the excitatory and the inhibitory signal was modified by decreasing the gain of the local inhibitory neurons within an assembly. In integrate-and-fire neurons such a gain change is equivalent to reducing the strength of all synapses onto inhibitory neurons by 75%. These values were chosen to allow for high firing rates in the presence of a stimulus.

To characterize the global state of the network we monitored individual spike trains, the population firing rate (the average of firing rates across the network), and the population rate's standard deviation σ_{Rate}, as well as average membrane potentials, and interspike intervals (ISIs) (Vogels and Abbott, 2009). The irregular asynchronous network activity that is thought to mimic cortical dynamics has a roughly constant population firing rate with low spiking correlation values and coefficients of variation of the interspike intervals (ISI CVs) near 1. The ISI CV for a neuron is the ratio of the standard deviation of the ISI distribution and its mean. ISI CV values close to zero indicate regular spiking patterns, values near 1 indicate irregular spiking, and values larger than 1 indicate, in our simulations, 'burstiness' in the firing pattern. All simulations were programmed in C.

To produce the presented results we used the following protocol:

1) We initialized the network with the above-described parameters and uniform synaptic weights, with the exception of neurons within the four pre-defined assemblies, which had ten-fold strengthened excitatory weights between neurons of the same assembly. Firing rates of both, background and assembly neurons were initially elevated. Inhibitory synaptic plasticity quickly established a target rate of ρ_0 = 6 Hz by increasing the inhibitory weights onto each neuron (Vogels et al., 2011). After the target rate was reached, ISP continued to adjust the weight-structure of inhibitory synapses until the best possible 'detailed balance' was reached and the network reached a state in which the spiking behaviour of the neurons became virtually indistinguishable from each other (Fig. 6E, Fig. S5B) (Hennequin et al., 2014; Vogels and Abbott, 2009).

2) We could activate any assembly individually by decreasing the gain, i.e. in this case, the efficacy of all incoming synapses, of the local inhibitory neurons within the group (Fig. 6A) (Vogels and Abbott, 2009). The decrease in inhibitory activity disturbed the EI balance of the chosen assembly in favour of excitation and inhibition and the assembly began to fire at high rates until the gain of the inhibitory cell population was set back to normal. We can do this either periodically as in Fig. S5 (every 40s) or intermittently, to avoid crosstalk between the (spike-dependent) plasticity rule and the high firing rates during stimulation. Such cross-talk had little effect on the phenomenon itself, but could lead to firing rates slightly lower than target rate ρ_0 as ISP would attempt to quench the periodically stimulated high firing rates.

3) To simulate association between two representations, we strengthened the excitatory connections from one assembly to neurons of any type within the other assembly by five-fold. As a result, the firing rates of all involved neurons increased, but as before, ISP quenched the excess firing rates, and then continually re-adjusted the strengths of the relevant inhibitory synapses. When we stimulated an assembly via gain reduction of the appropriate inhibitory neurons as in step 2, *before* detailed balance was reached, the increased firing rates in one assembly supplied enough excitatory current to its paired neuron group that both assemblies fired at high rates (Fig. 6B, Fig. S5B, cf.Vogels et al., 2011). Additionally, there could be spontaneous self-sustained co-activation of unrelated assemblies in some of the trials because the net excitation initially far exceeded the inhibition, leading to transients in the blue and yellow curves in Fig. 6C. After ISP had sufficiently strengthened disynaptic feed forward inhibition so to counteract the excess excitation (Fig. S4C), this co-activation was reduced to a brief activity transient before inhibitory firing silenced the response (Fig. 6C).

We could re-evoke the response-coupling of two synaptically linked assemblies by subtly reducing the ambient tone of inhibitory activity, i.e. by reducing the efficacy of all inhibitory synapses in the network by 15%. This led to only moderate changes in baseline firing rates (Fig. S6). For targeted stimulation of one assembly, the change in inhibitory baseline efficacy produced co-activation of assembly patterns as before, because even moderately disadvantaged inhibition within the assemblies could not balance the additional excitatory activity it received from its paired neuron group (Fig. 6D, Fig. S4D, cf. Vogels and Abbott, 2007).

Supplemental References:

Bednařík, P., Tkáč, I., Giove, F., DiNuzzo, M., Deelchand, D.K., Emir, U.E., Eberly, L.E., and Mangia, S. (2015). Neurochemical and BOLD responses during neuronal activation measured in the human visual cortex at 7 Tesla. J. Cereb. Blood Flow Metab. *35*, 601–610.

Emir, U.E., Tuite, P.J., and Öz, G. (2012). Elevated Pontine and Putamenal GABA Levels in Mild-Moderate Parkinson Disease Detected by 7 Tesla Proton MRS. PLoS ONE *7*, e30918.

Govindaraju, V., Young, K., and Maudsley, A.A. (2000). Proton NMR chemical shifts and coupling constants for brain metabolites. NMR Biomed. *13*, 129–153.

Hennequin, G., Vogels, T.P., and Gerstner, W. (2014). Optimal Control of Transient Dynamics in Balanced Networks Supports Generation of Complex Movements. Neuron *82*, 1394–1406.

Large, M-E., Aldcroft A., and Vilis T. (2007). Task-related laterality effects in the lateral occiptal complex. Brain Research *1128*, 130-138.

Natt, O., Bezkorovaynyy, V., Michaelis, T., and Frahm, J. (2005). Use of phased array coils for a determination of absolute metabolite concentrations. Magn. Reson. Med. Off. J. Soc. Magn. Reson. Med. Soc. Magn. Reson. Med. *53*, 3–8.

Rossum, M.C.W. van, Bi, G.Q., and Turrigiano, G.G. (2000). Stable Hebbian Learning from Spike Timing-Dependent Plasticity. J. Neurosci. *20*, 8812–8821.

Rossum, M.C.W. van, Turrigiano, G.G., and Nelson, S.B. (2002). Fast Propagation of Firing Rates through Layered Networks of Noisy Neurons. J. Neurosci. *22*, 1956–1966.

Soher B.J., Semanchuk P., Todd D., Steinberg J., & Young K. (2011). VeSPA: Integrated applications for RF pulse design, spectral simulation and MRS data analysis. Proc. Int. Soc. Magn. Reson. Med.

Tkac I. (2008). Refinement of simulated basis set for LCModel analysis. Proc. 16th Annu. Meet. ISMRM Tor. Can.

Vogels, T.P., and Abbott, L.F. (2005). Signal Propagation and Logic Gating in Networks of Integrate-and-Fire Neurons. J. Neurosci. *25*, 10786–10795.
